# Supplementary figures and images for: AMPK activation by glycogen expenditure primes the exit of naïve pluripotency
Source: EMBO Rep. 2025 Feb 17;26(6):1504–27. doi: 10.1038/s44319-025-00384-x (PMC11933299; doi:10.1038/s44319-025-00384-x)

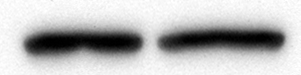

Supplement: Supplementary file 4 — Source data Fig. 2 [file 44319_2025_384_MOESM4_ESM.zip › Figure 2/Fig. 2D/bactin.tif]

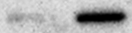

Supplement: Supplementary file 4 — Source data Fig. 2 [file 44319_2025_384_MOESM4_ESM.zip › Figure 2/Fig. 2D/pAcc.tif]

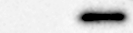

Supplement: Supplementary file 4 — Source data Fig. 2 [file 44319_2025_384_MOESM4_ESM.zip › Figure 2/Fig. 2D/pAmpk.tif]

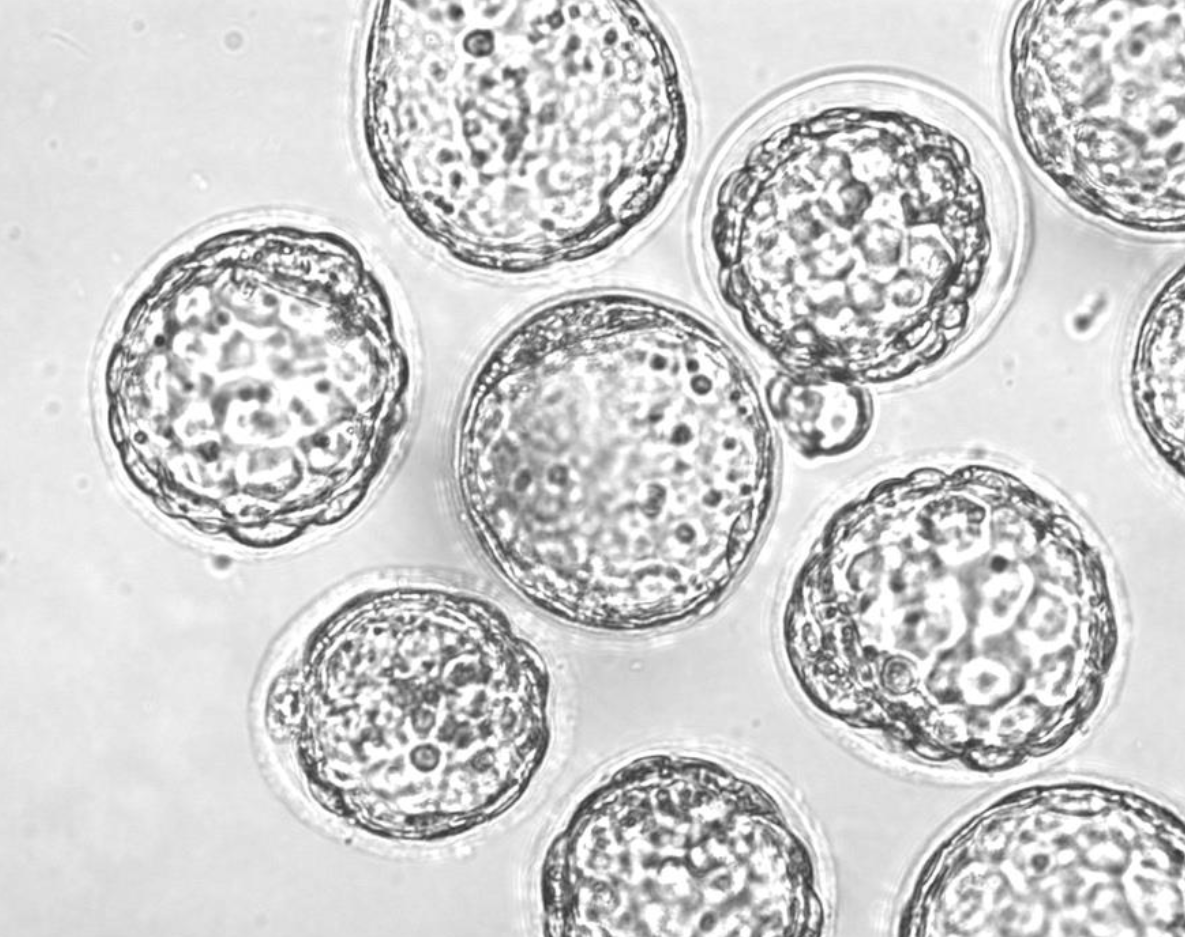

Supplement: Supplementary file 4 — Source data Fig. 2 [file 44319_2025_384_MOESM4_ESM.zip › Figure 2/Fig. 2I/BF.tif]

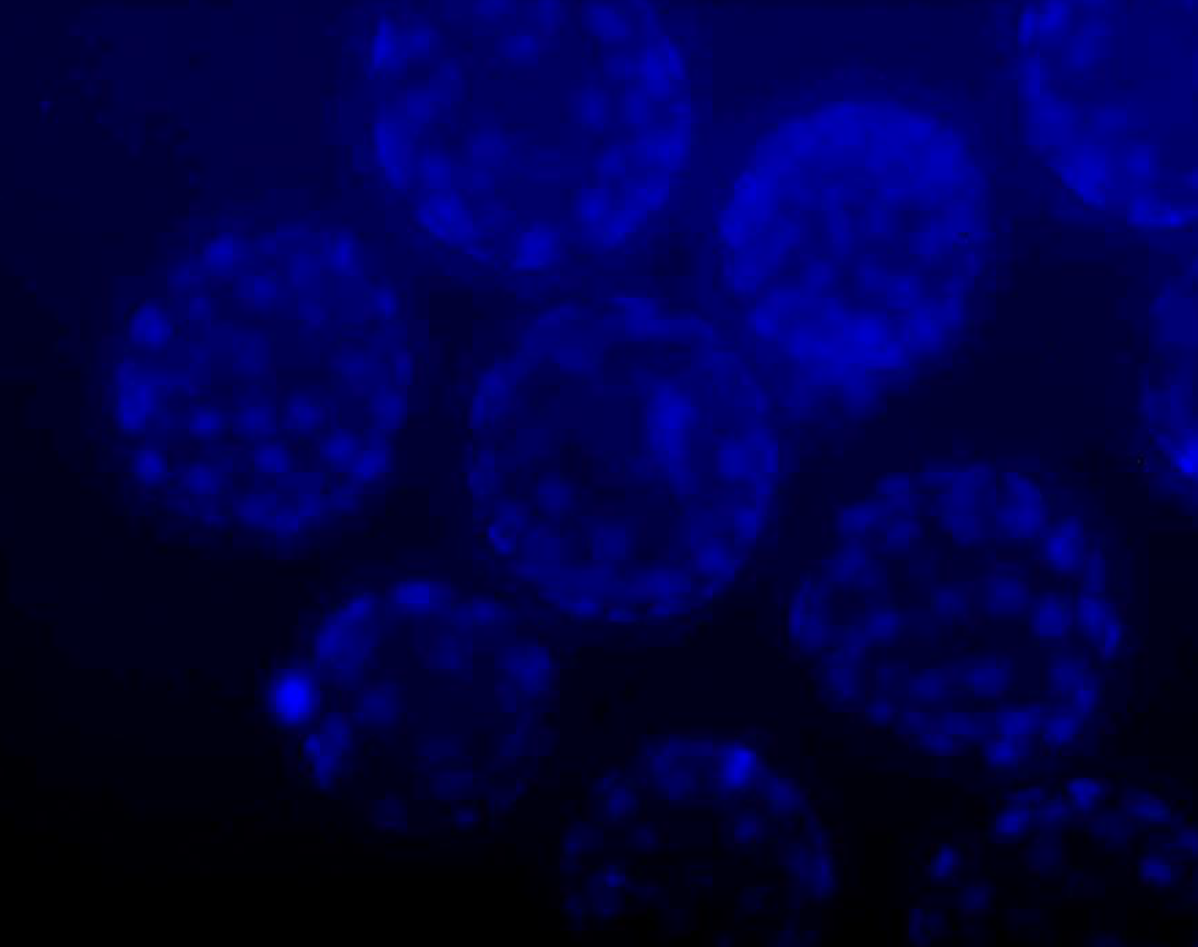

Supplement: Supplementary file 4 — Source data Fig. 2 [file 44319_2025_384_MOESM4_ESM.zip › Figure 2/Fig. 2I/Hoech.tif]

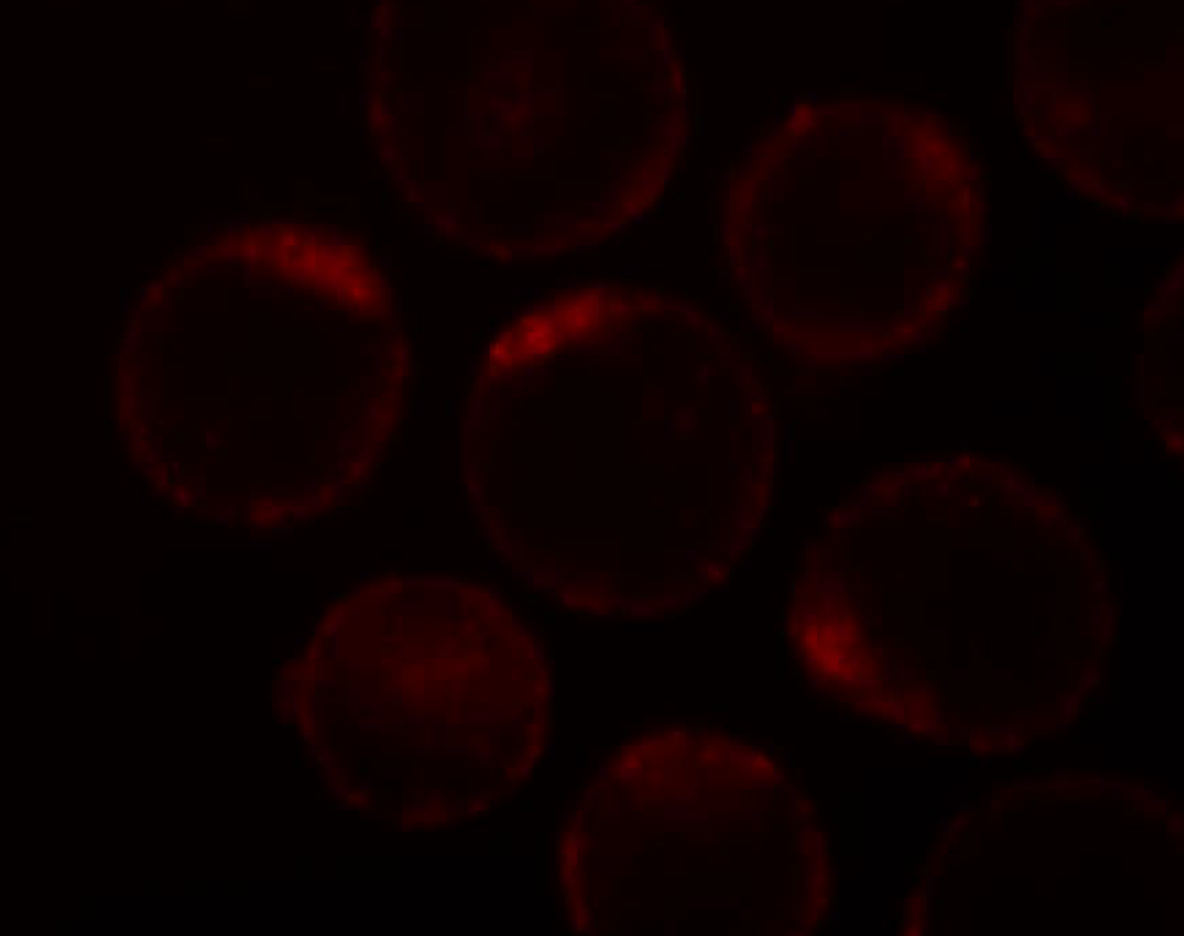

Supplement: Supplementary file 4 — Source data Fig. 2 [file 44319_2025_384_MOESM4_ESM.zip › Figure 2/Fig. 2I/NR.tif]

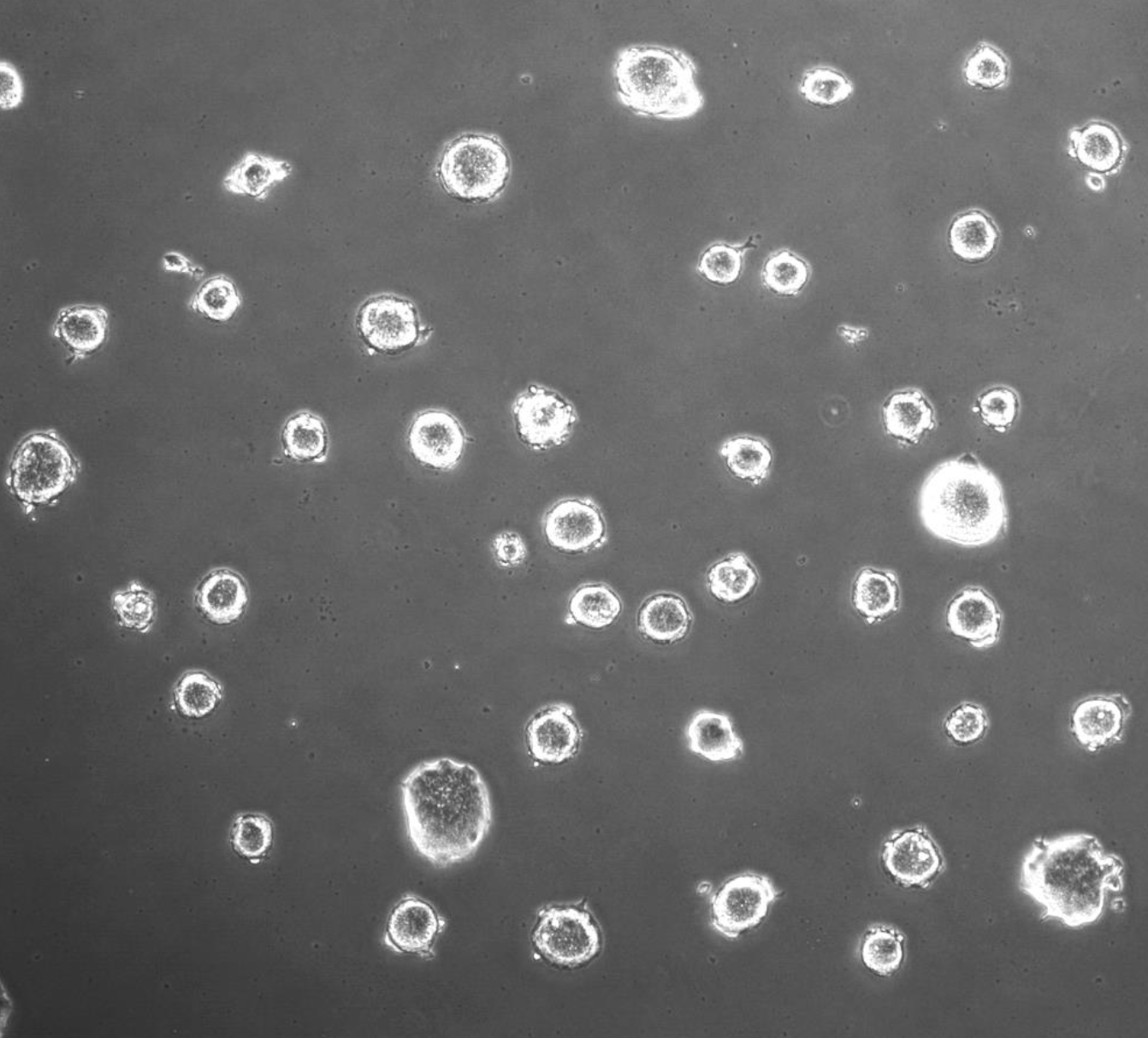

Supplement: Supplementary file 5 — Source data Fig. 3 [file 44319_2025_384_MOESM5_ESM.zip › Figure 3/Fig. 3A/Naive(+).tif]

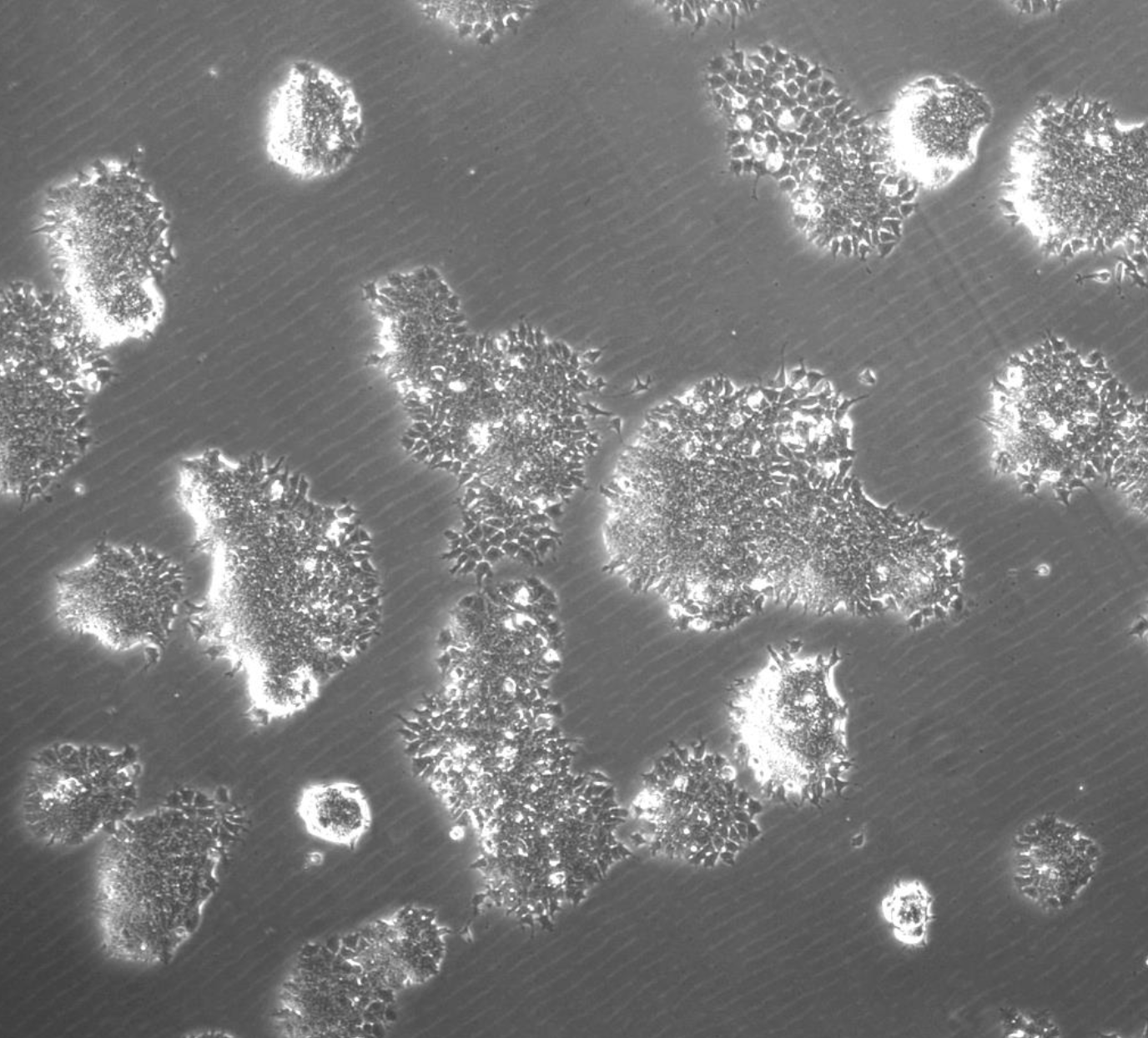

Supplement: Supplementary file 5 — Source data Fig. 3 [file 44319_2025_384_MOESM5_ESM.zip › Figure 3/Fig. 3A/Naive(-).tif]

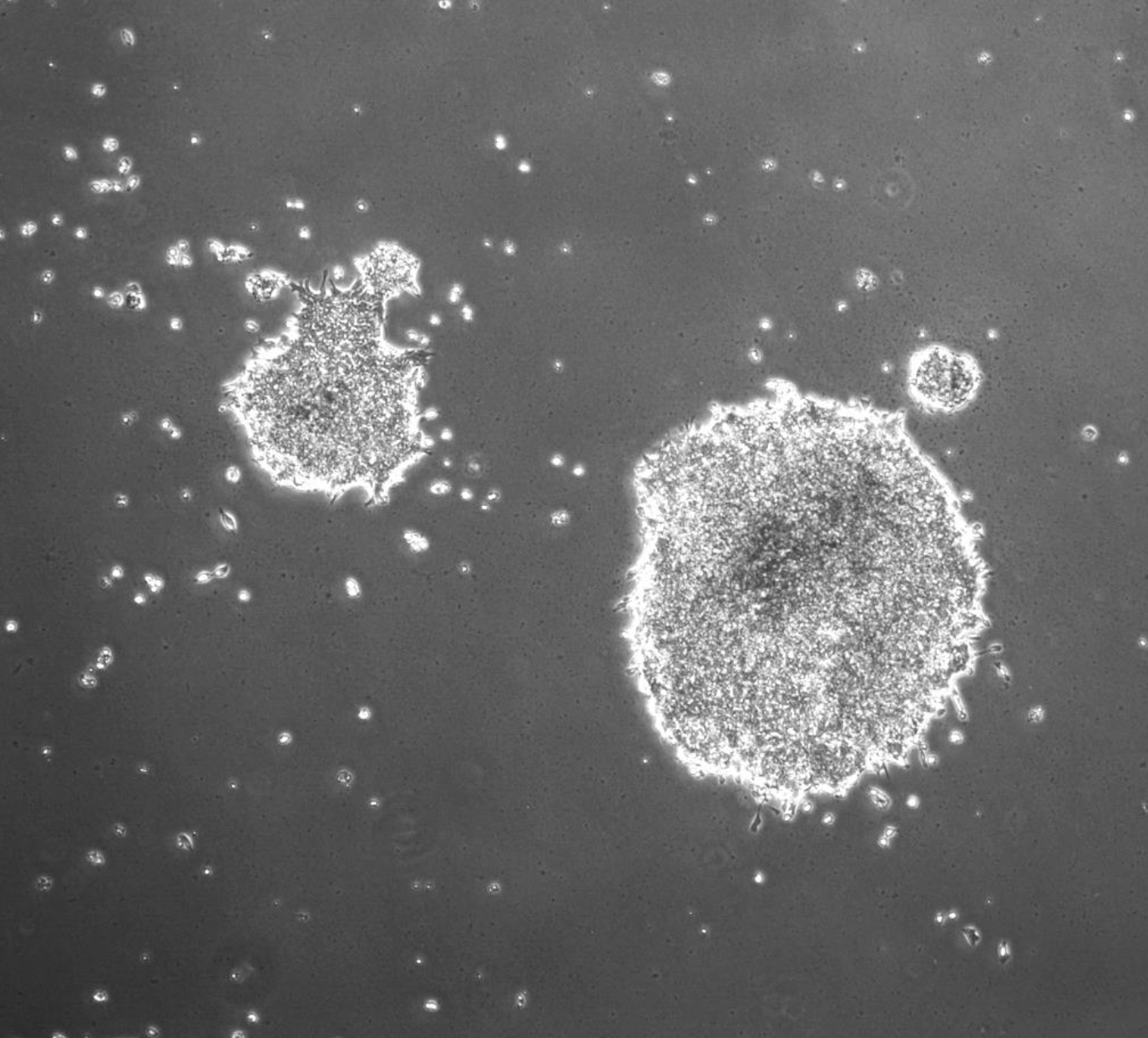

Supplement: Supplementary file 5 — Source data Fig. 3 [file 44319_2025_384_MOESM5_ESM.zip › Figure 3/Fig. 3A/Primed(+).tif]

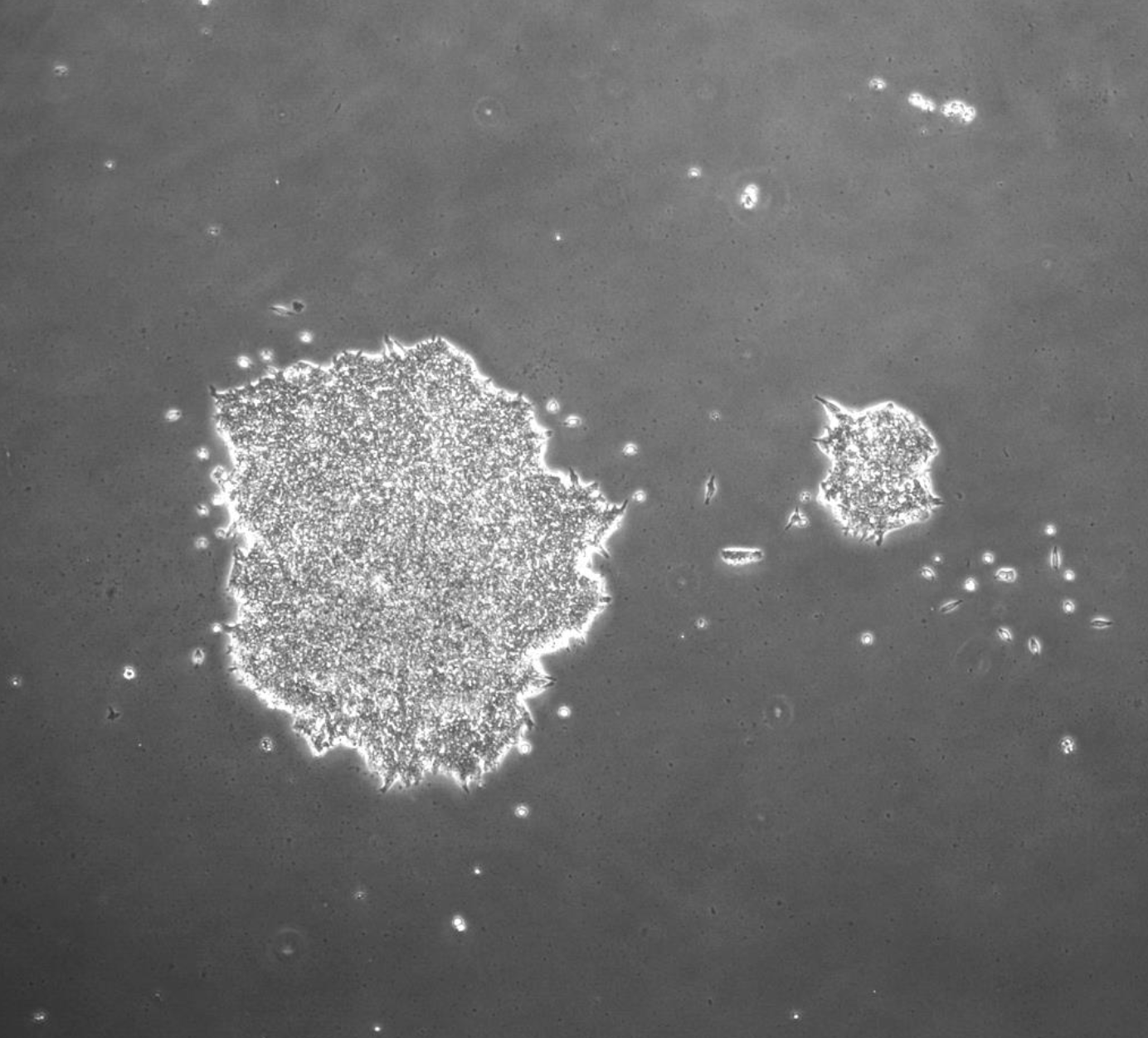

Supplement: Supplementary file 5 — Source data Fig. 3 [file 44319_2025_384_MOESM5_ESM.zip › Figure 3/Fig. 3A/Primed(-).tif]

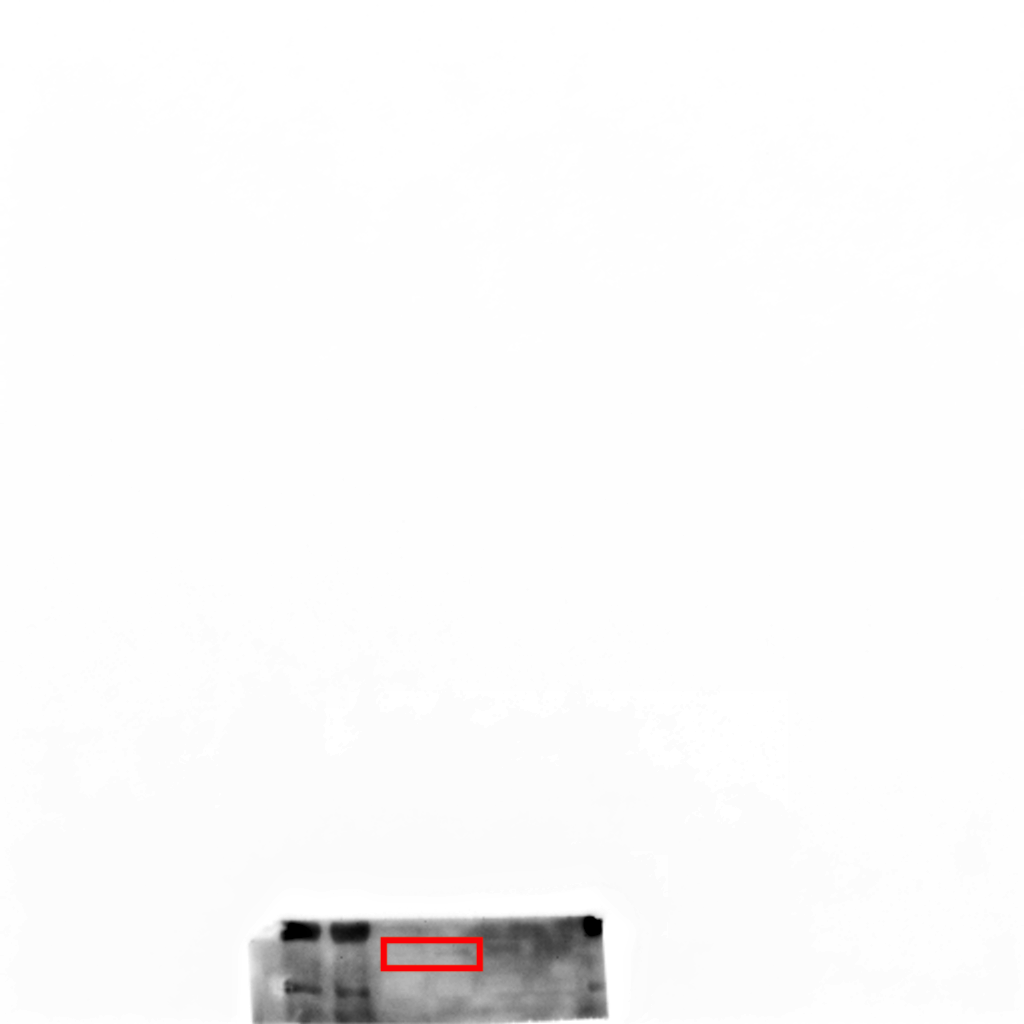

Supplement: Supplementary file 5 — Source data Fig. 3 [file 44319_2025_384_MOESM5_ESM.zip › Figure 3/Fig. 3B/pAmpka.TIF]

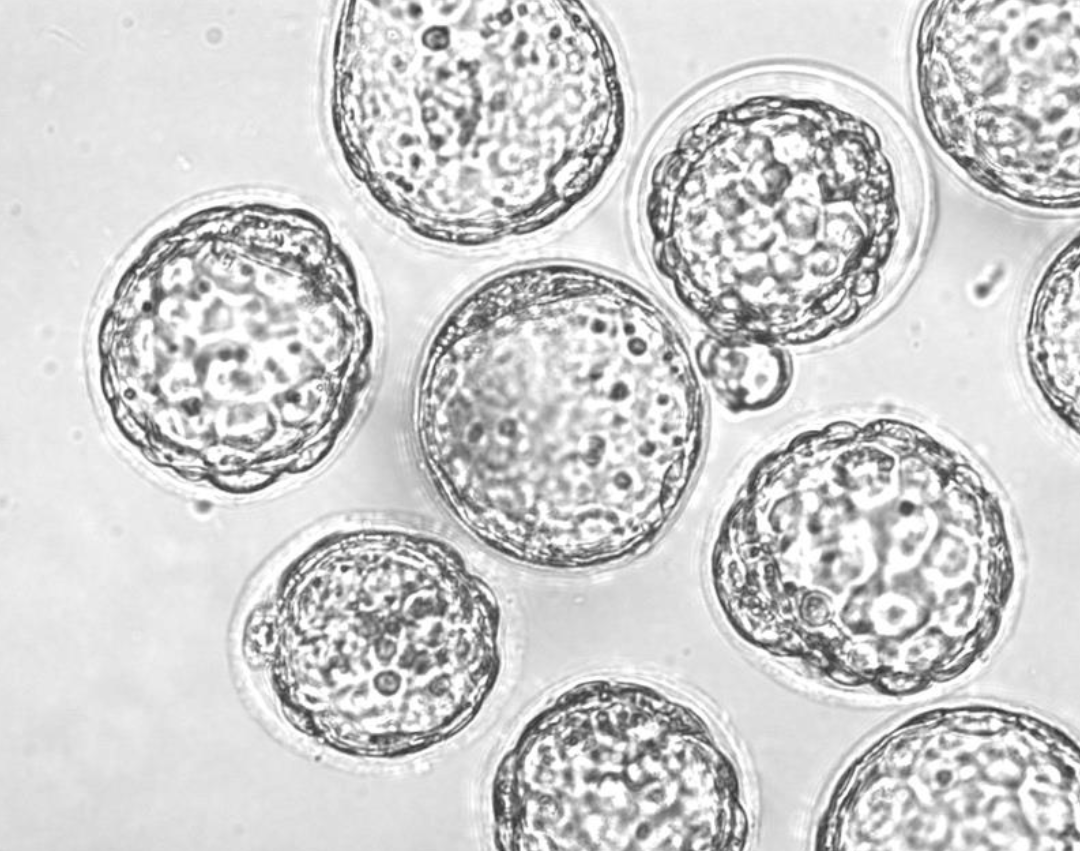

Supplement: Supplementary file 5 — Source data Fig. 3 [file 44319_2025_384_MOESM5_ESM.zip › Figure 3/Fig. 3C/BF.tif]

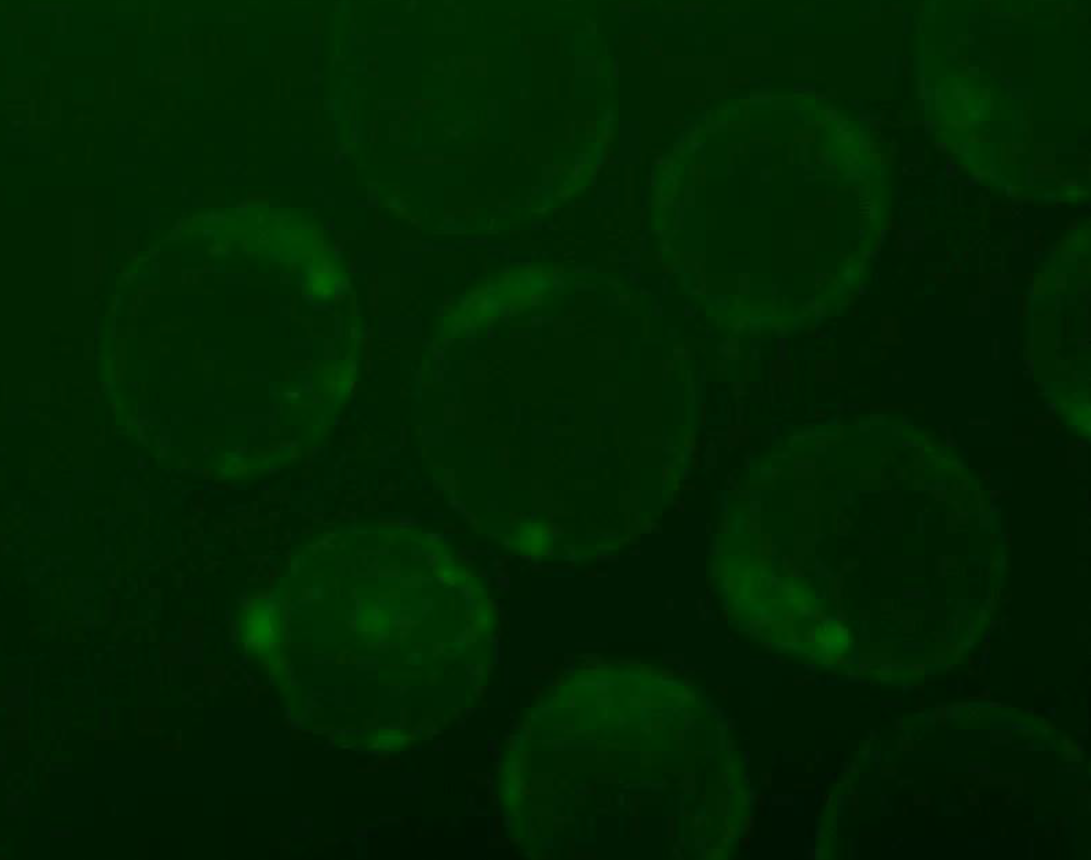

Supplement: Supplementary file 5 — Source data Fig. 3 [file 44319_2025_384_MOESM5_ESM.zip › Figure 3/Fig. 3C/CDg4.tif]

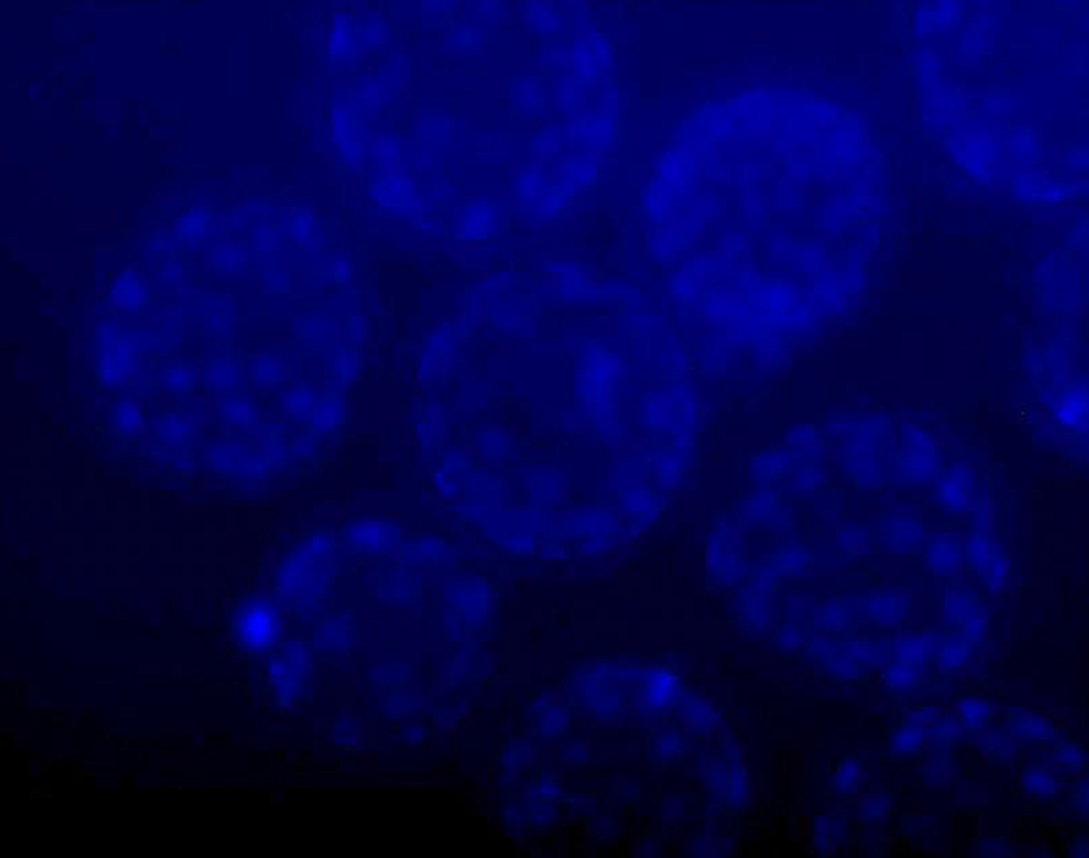

Supplement: Supplementary file 5 — Source data Fig. 3 [file 44319_2025_384_MOESM5_ESM.zip › Figure 3/Fig. 3C/Hoech.tif]

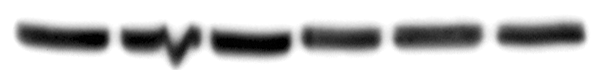

Supplement: Supplementary file 5 — Source data Fig. 3 [file 44319_2025_384_MOESM5_ESM.zip › Figure 3/Fig. 3D/atubulin]

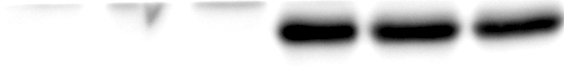

Supplement: Supplementary file 5 — Source data Fig. 3 [file 44319_2025_384_MOESM5_ESM.zip › Figure 3/Fig. 3D/oct4.tif]

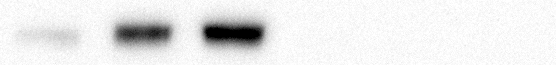

Supplement: Supplementary file 5 — Source data Fig. 3 [file 44319_2025_384_MOESM5_ESM.zip › Figure 3/Fig. 3D/pAcc.tif]

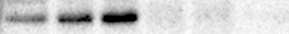

Supplement: Supplementary file 5 — Source data Fig. 3 [file 44319_2025_384_MOESM5_ESM.zip › Figure 3/Fig. 3D/pAMPK.tif]

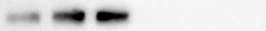

Supplement: Supplementary file 5 — Source data Fig. 3 [file 44319_2025_384_MOESM5_ESM.zip › Figure 3/Fig. 3D/Total Acc.tif]

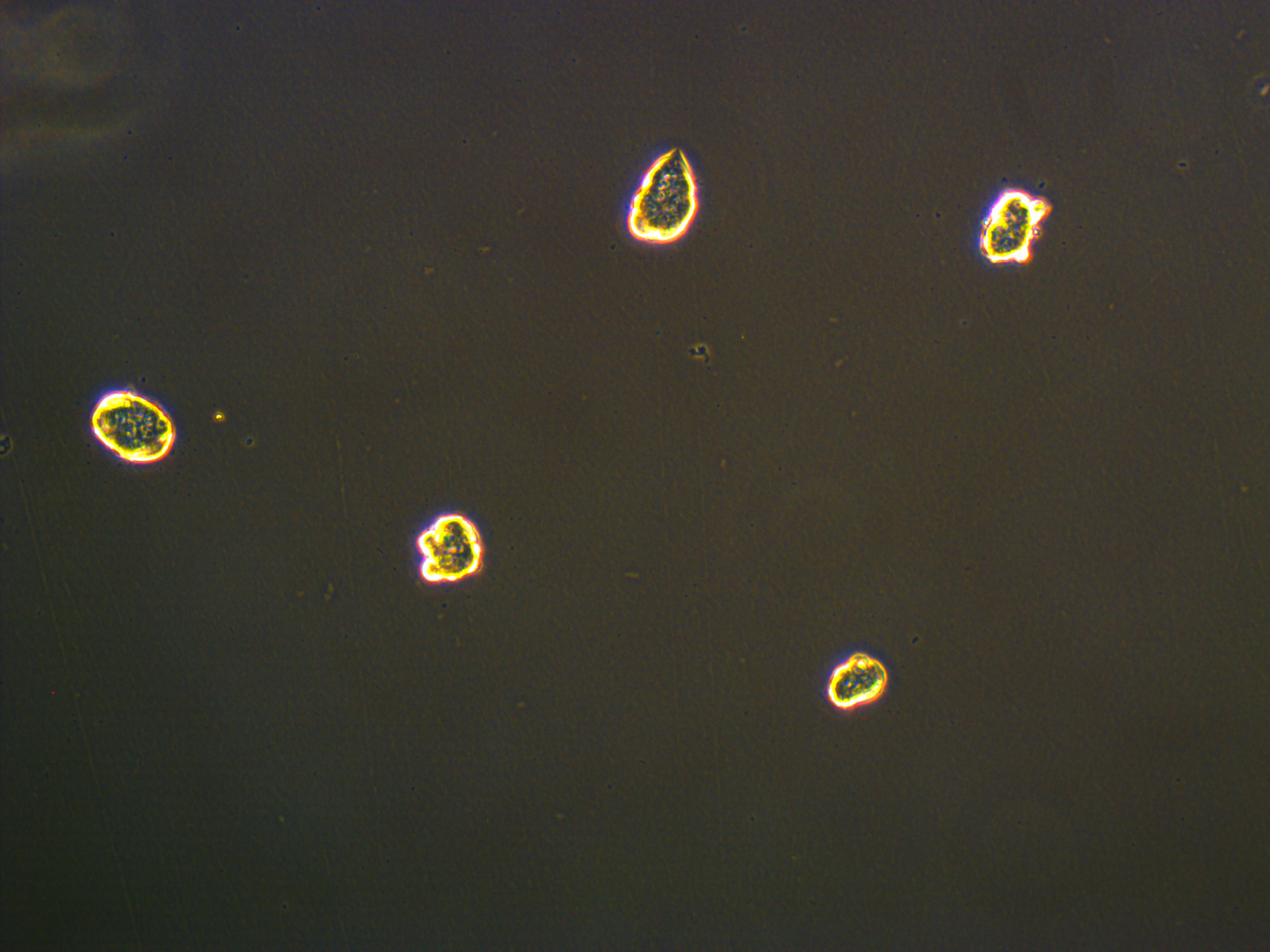

Supplement: Supplementary file 5 — Source data Fig. 3 [file 44319_2025_384_MOESM5_ESM.zip › Figure 3/Fig. 3F/AKO (+).tif]

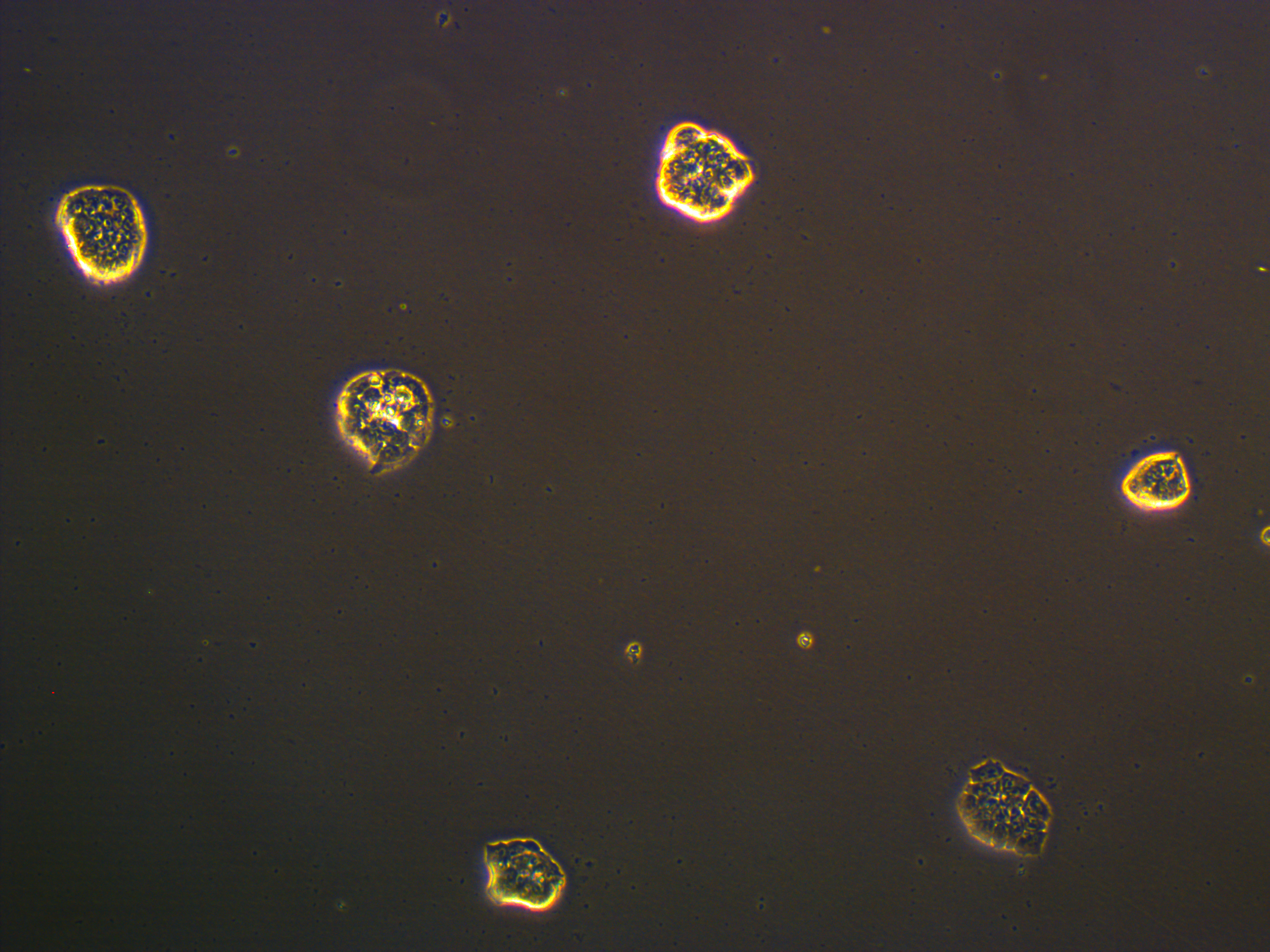

Supplement: Supplementary file 5 — Source data Fig. 3 [file 44319_2025_384_MOESM5_ESM.zip › Figure 3/Fig. 3F/AKO (-).tif]

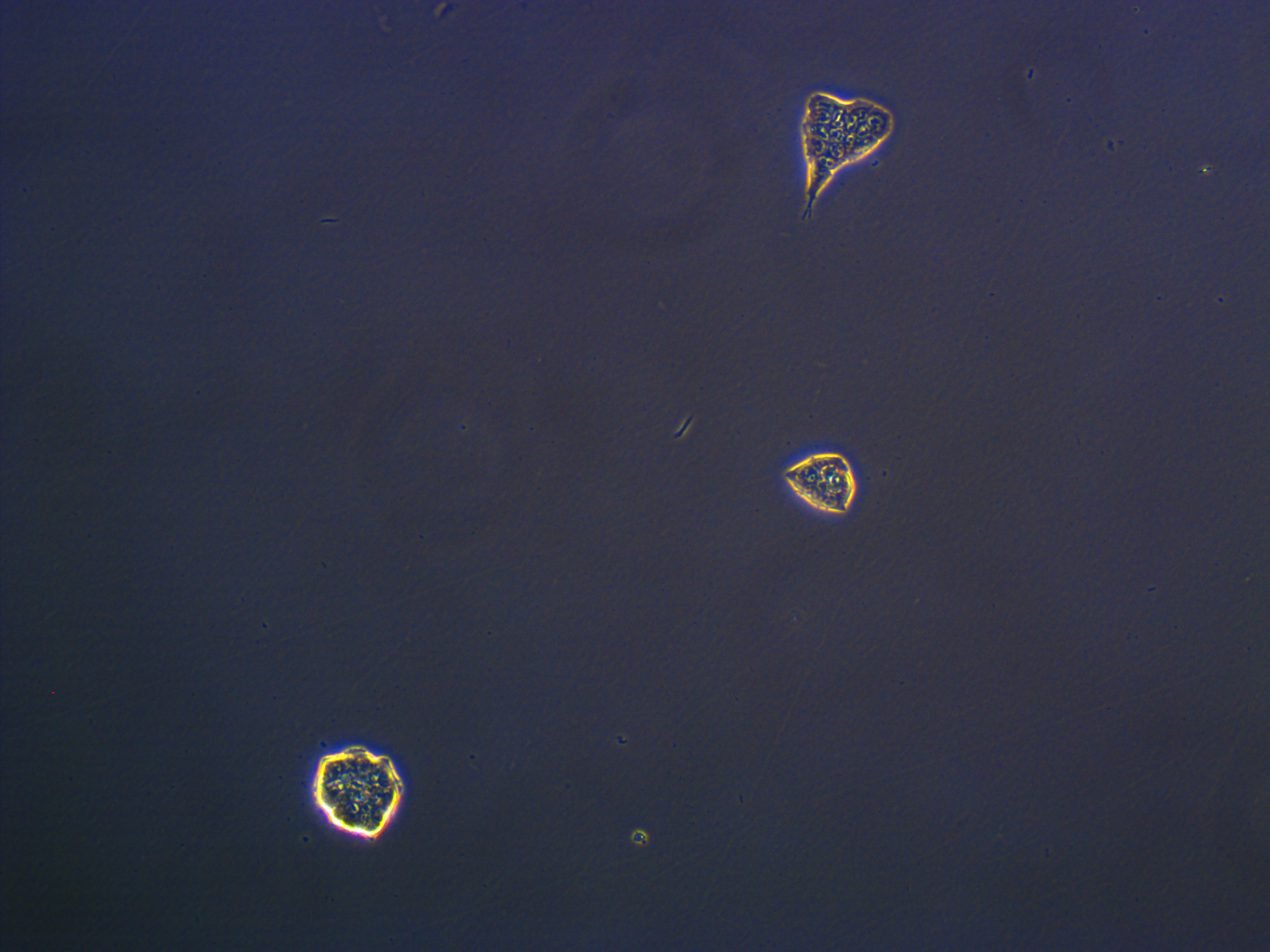

Supplement: Supplementary file 5 — Source data Fig. 3 [file 44319_2025_384_MOESM5_ESM.zip › Figure 3/Fig. 3F/Cont (+).tif]

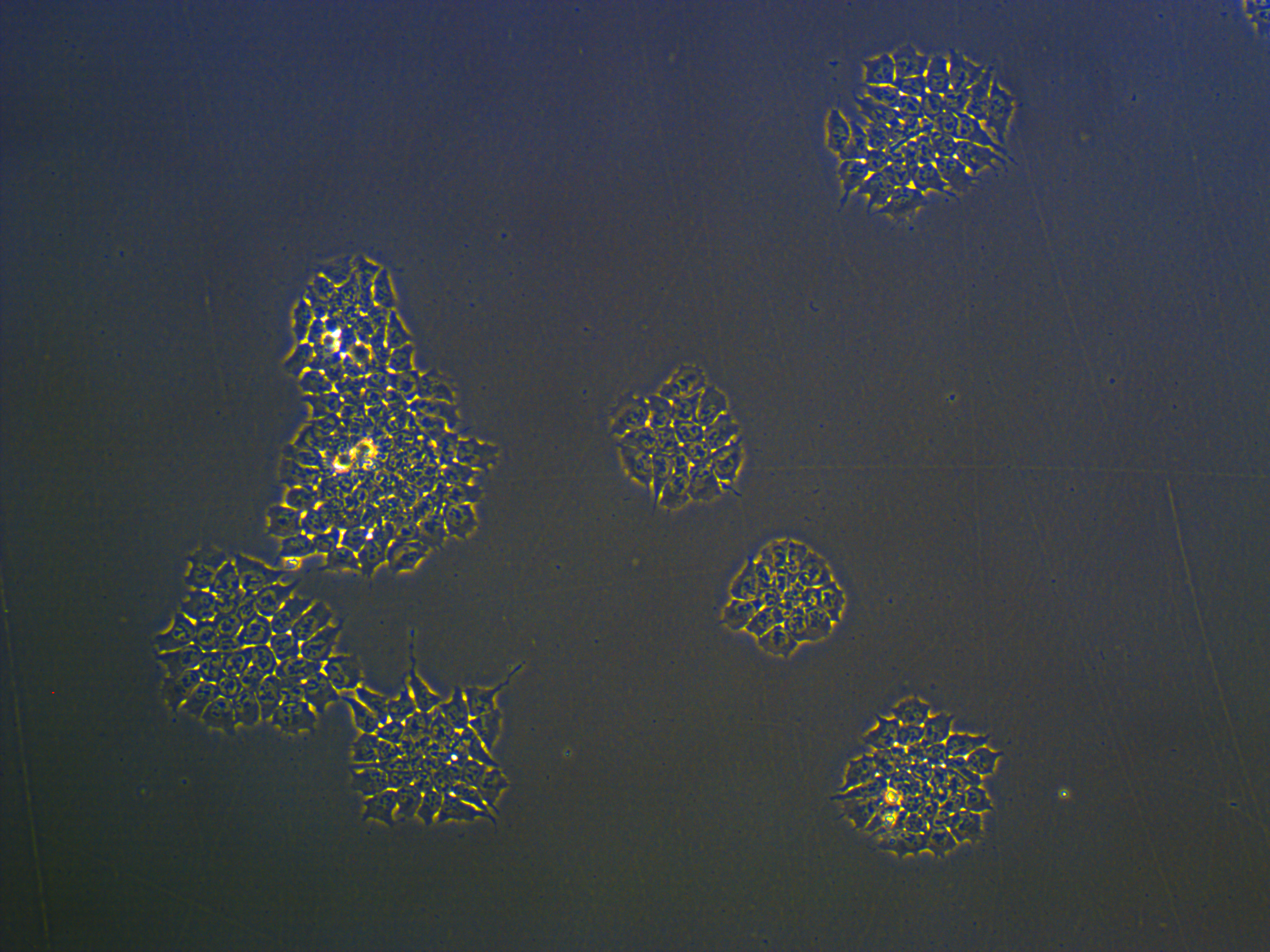

Supplement: Supplementary file 5 — Source data Fig. 3 [file 44319_2025_384_MOESM5_ESM.zip › Figure 3/Fig. 3F/Cont (-).tif]

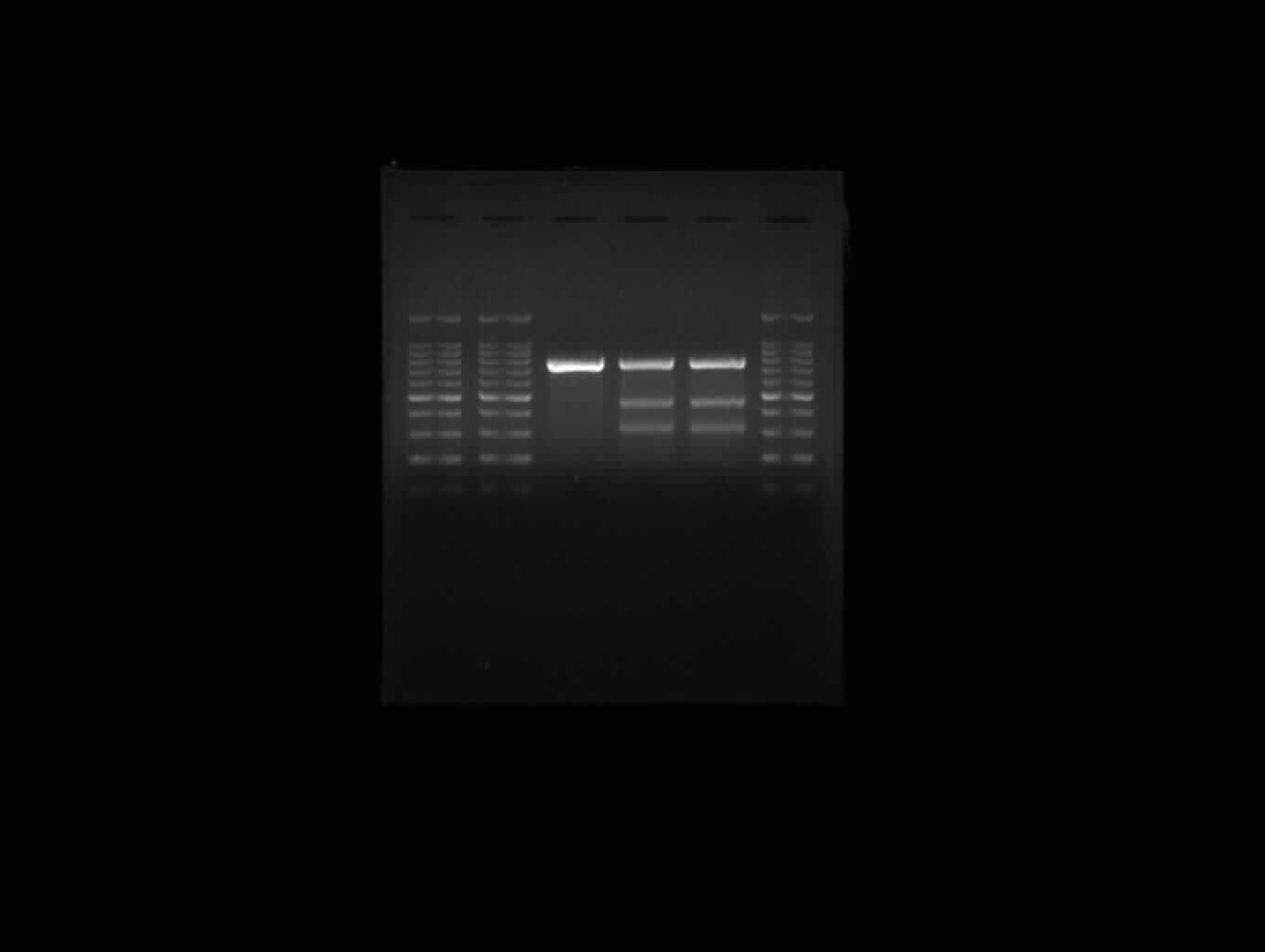

Supplement: Supplementary file 6 — Source data Fig. 4 [file 44319_2025_384_MOESM6_ESM.zip › Figure 4/Fig. 4B/200716_J1 Gys1 KO Pool (Re) T7E1.tiff]

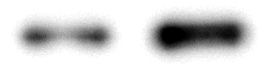

Supplement: Supplementary file 6 — Source data Fig. 4 [file 44319_2025_384_MOESM6_ESM.zip › Figure 4/Fig. 4E/Total Ampk.tif]

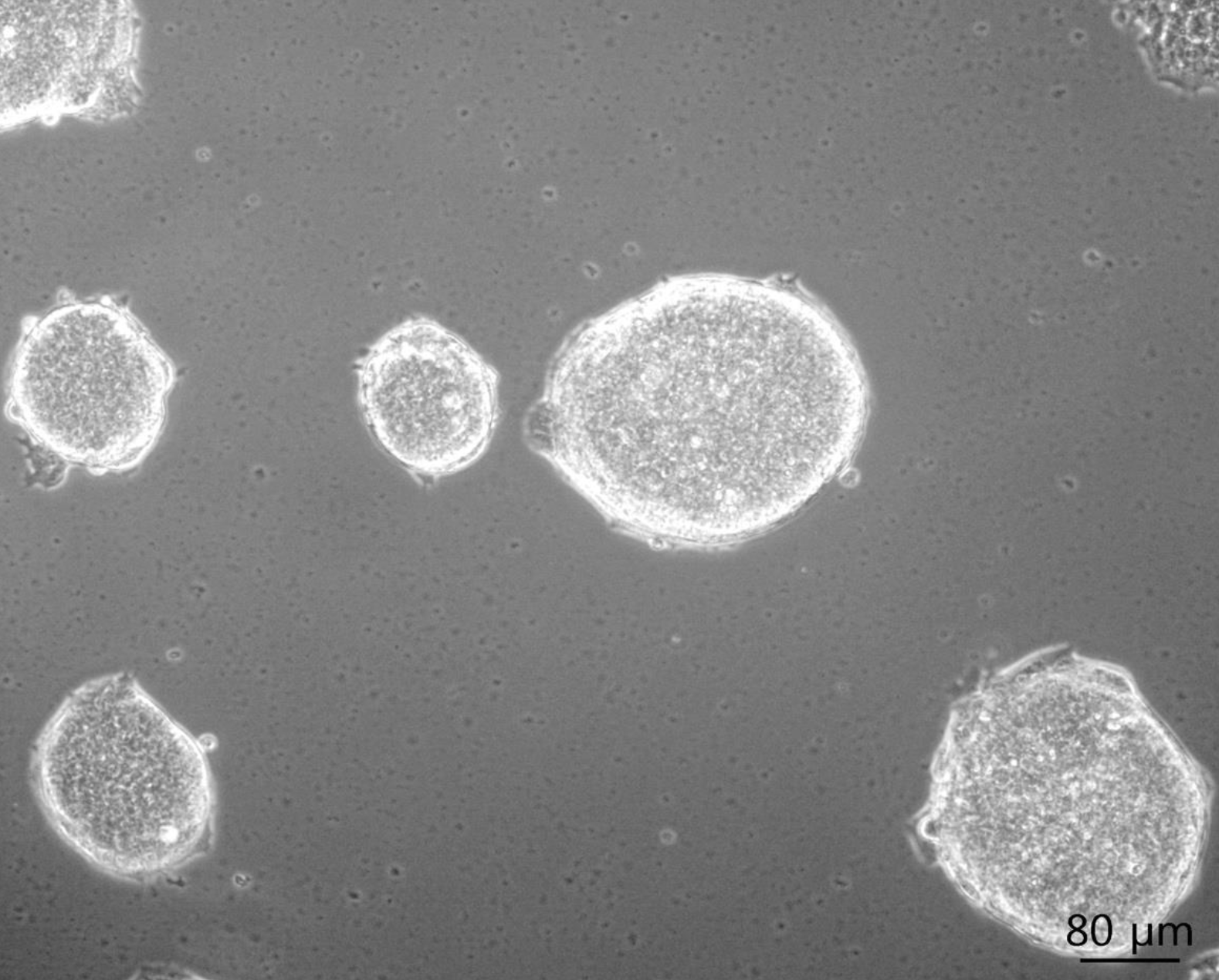

Supplement: Supplementary file 7 — Source data Fig. 5 [file 44319_2025_384_MOESM7_ESM.zip › Figure 5/Fig. 5A/Cont.tif]

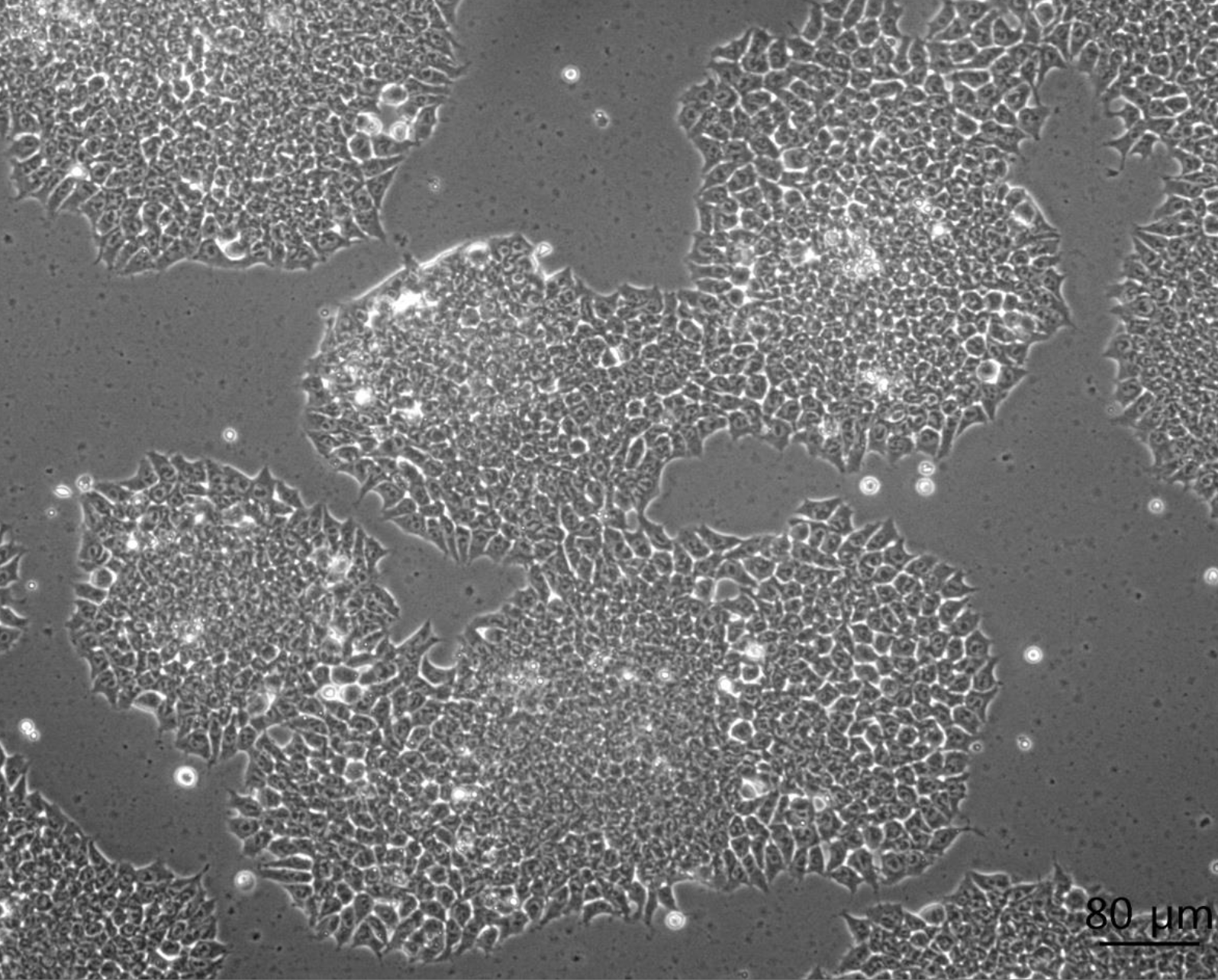

Supplement: Supplementary file 7 — Source data Fig. 5 [file 44319_2025_384_MOESM7_ESM.zip › Figure 5/Fig. 5A/GKO.tif]

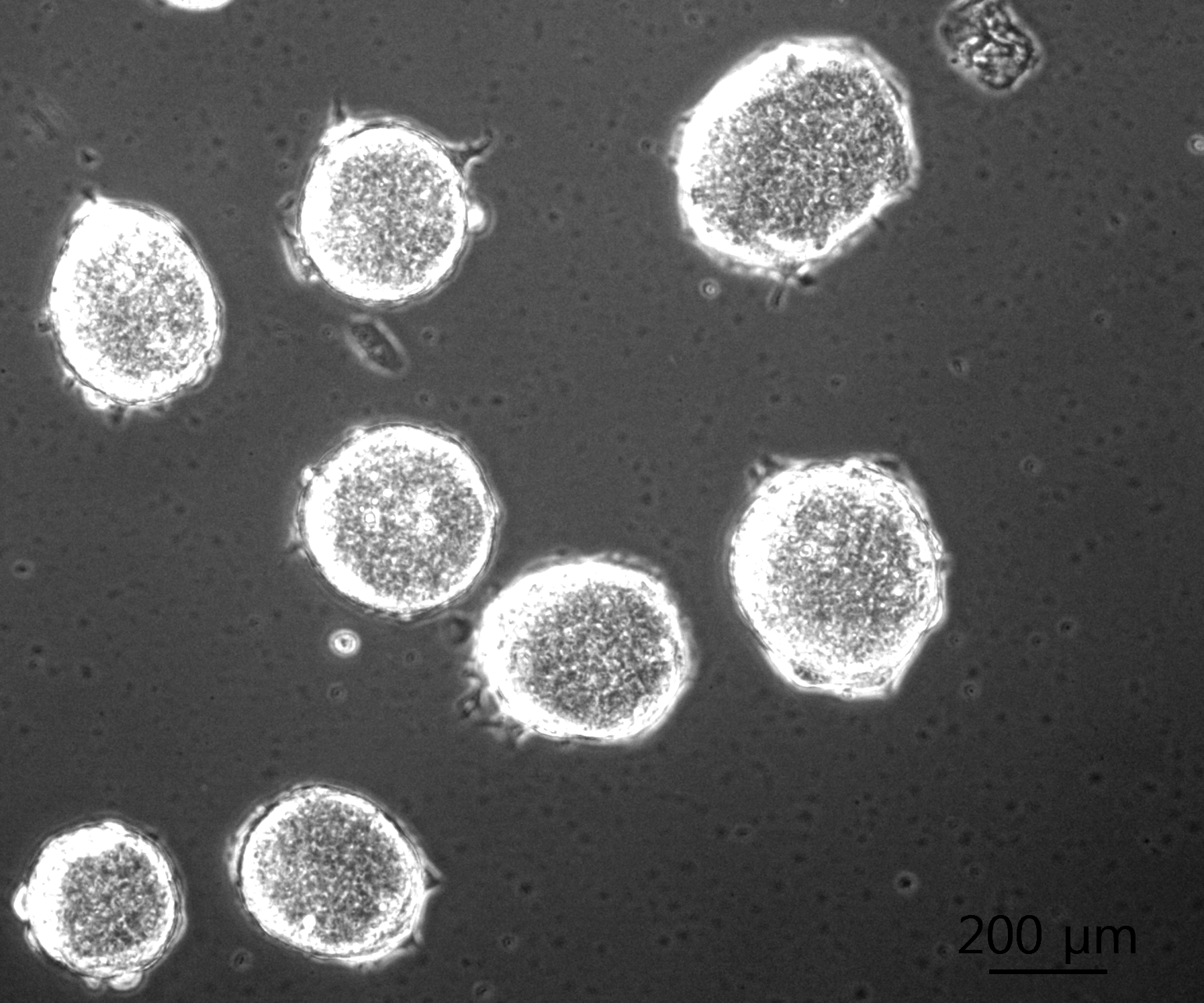

Supplement: Supplementary file 7 — Source data Fig. 5 [file 44319_2025_384_MOESM7_ESM.zip › Figure 5/Fig. 5E/J1 Cont_Mock.tif]

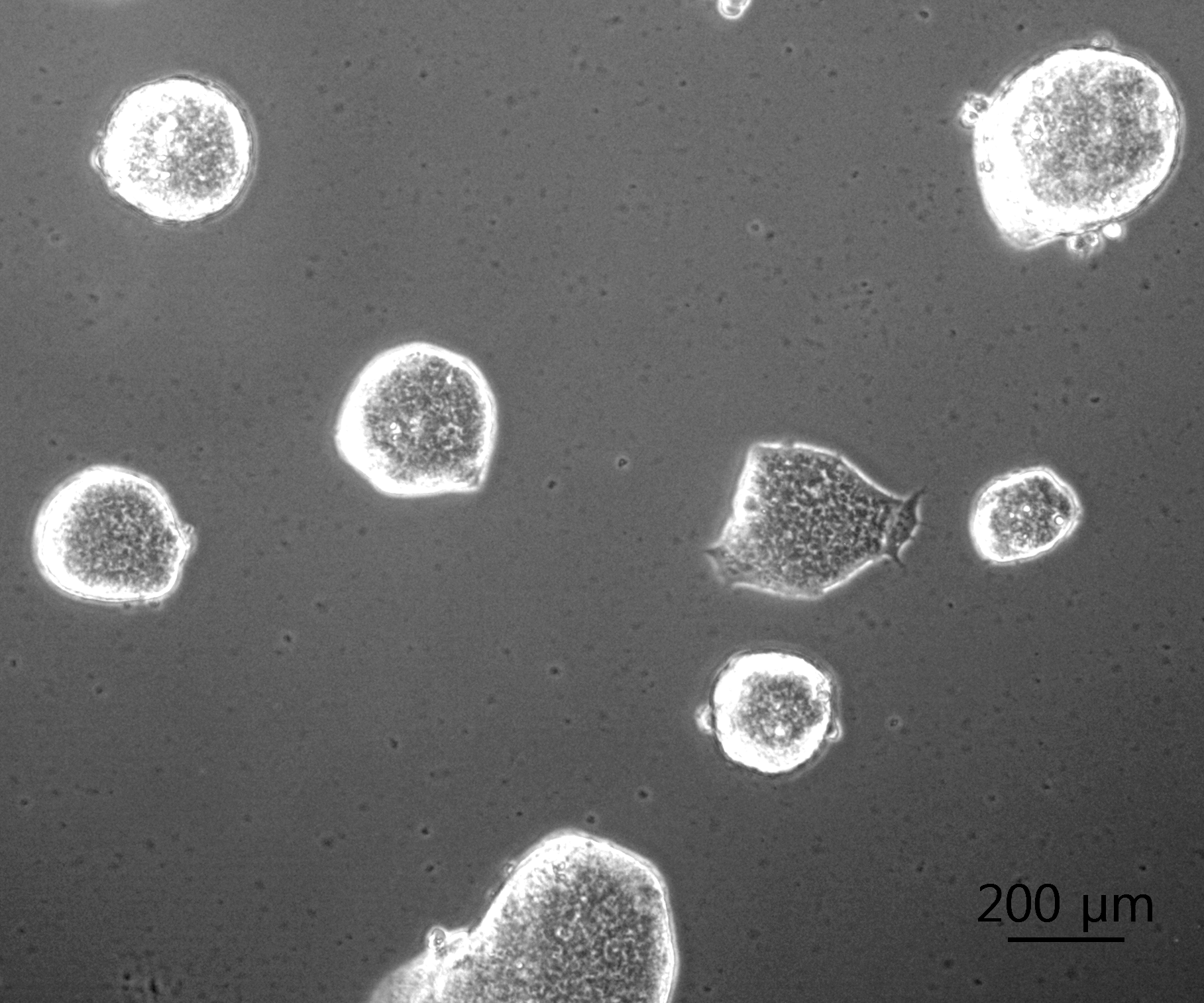

Supplement: Supplementary file 7 — Source data Fig. 5 [file 44319_2025_384_MOESM7_ESM.zip › Figure 5/Fig. 5E/J1 Cont_No Glc Day2.tif]

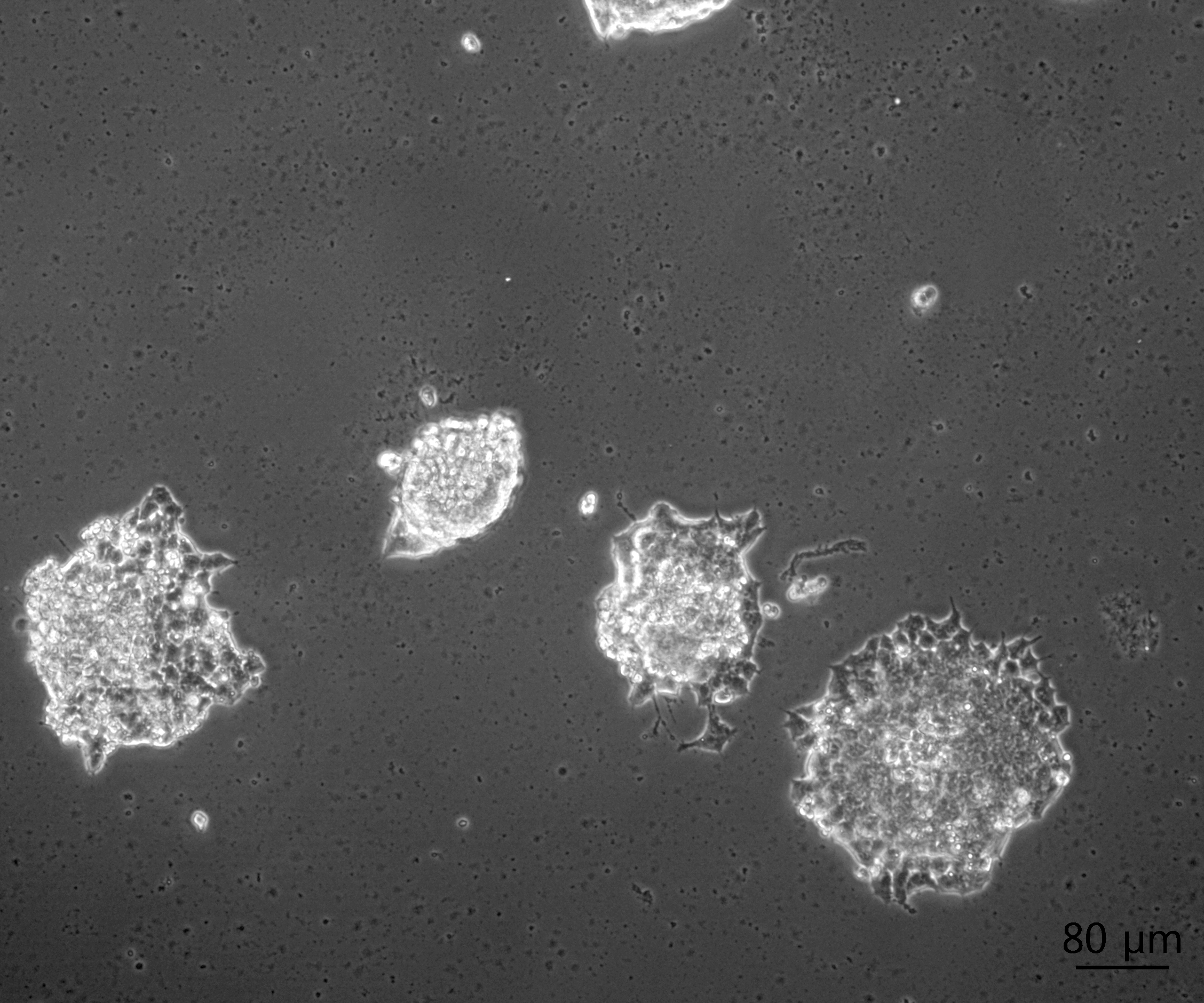

Supplement: Supplementary file 7 — Source data Fig. 5 [file 44319_2025_384_MOESM7_ESM.zip › Figure 5/Fig. 5E/J1 Cont_No Glc Day3.tif]

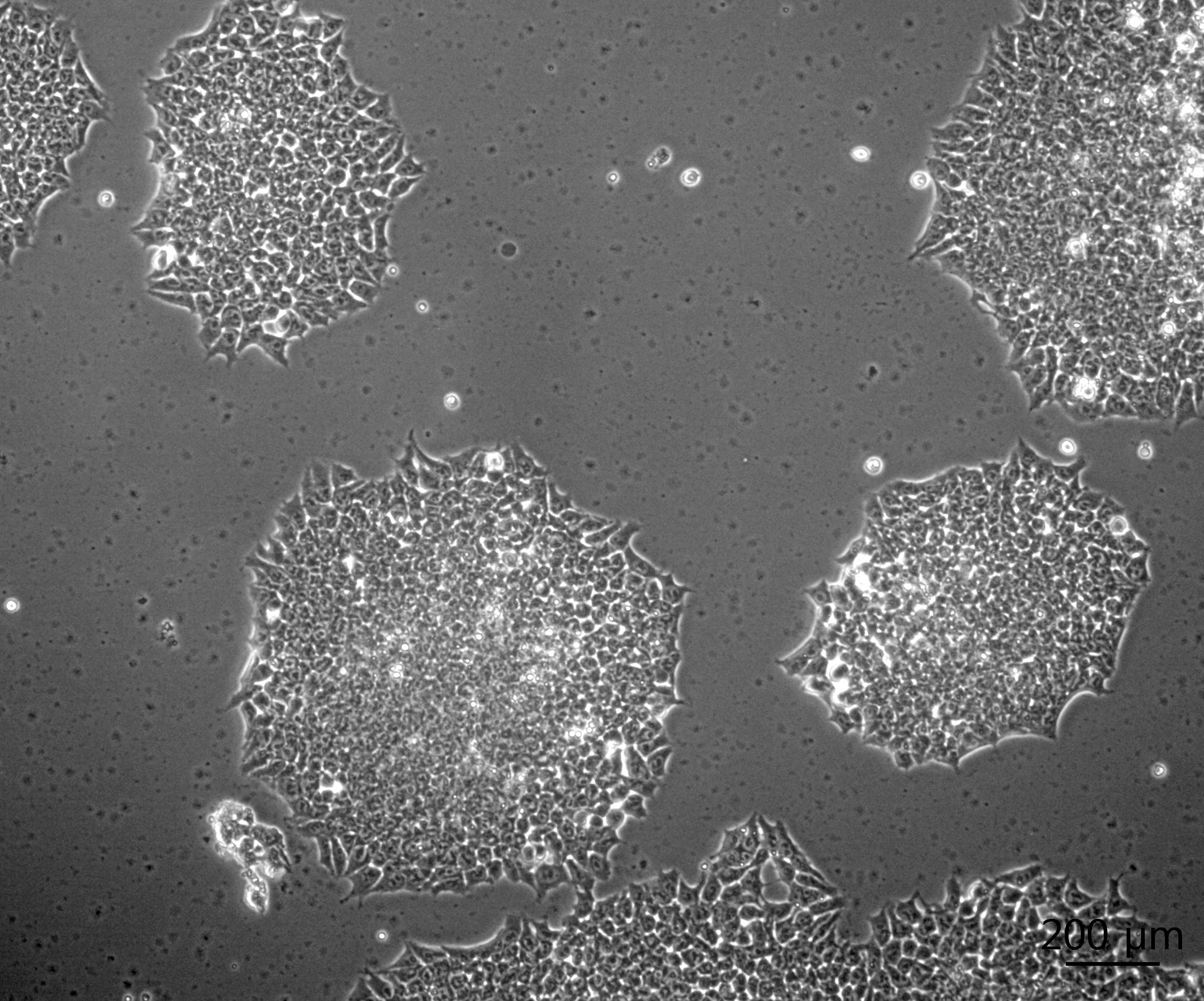

Supplement: Supplementary file 7 — Source data Fig. 5 [file 44319_2025_384_MOESM7_ESM.zip › Figure 5/Fig. 5E/J1 Gys1 KO_Mock.tif]

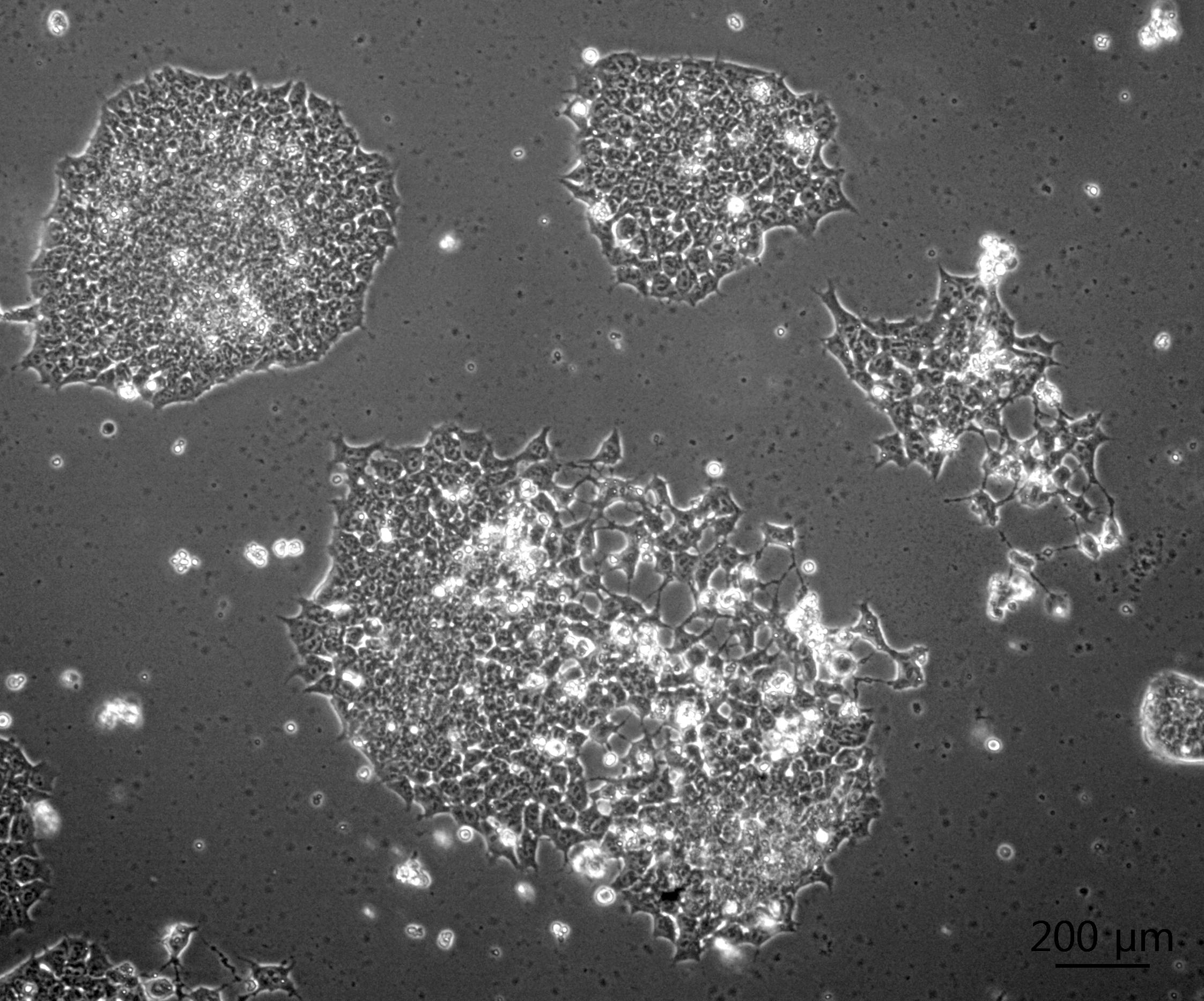

Supplement: Supplementary file 7 — Source data Fig. 5 [file 44319_2025_384_MOESM7_ESM.zip › Figure 5/Fig. 5E/J1 Gys1 KO_No Glc Day2.tif]

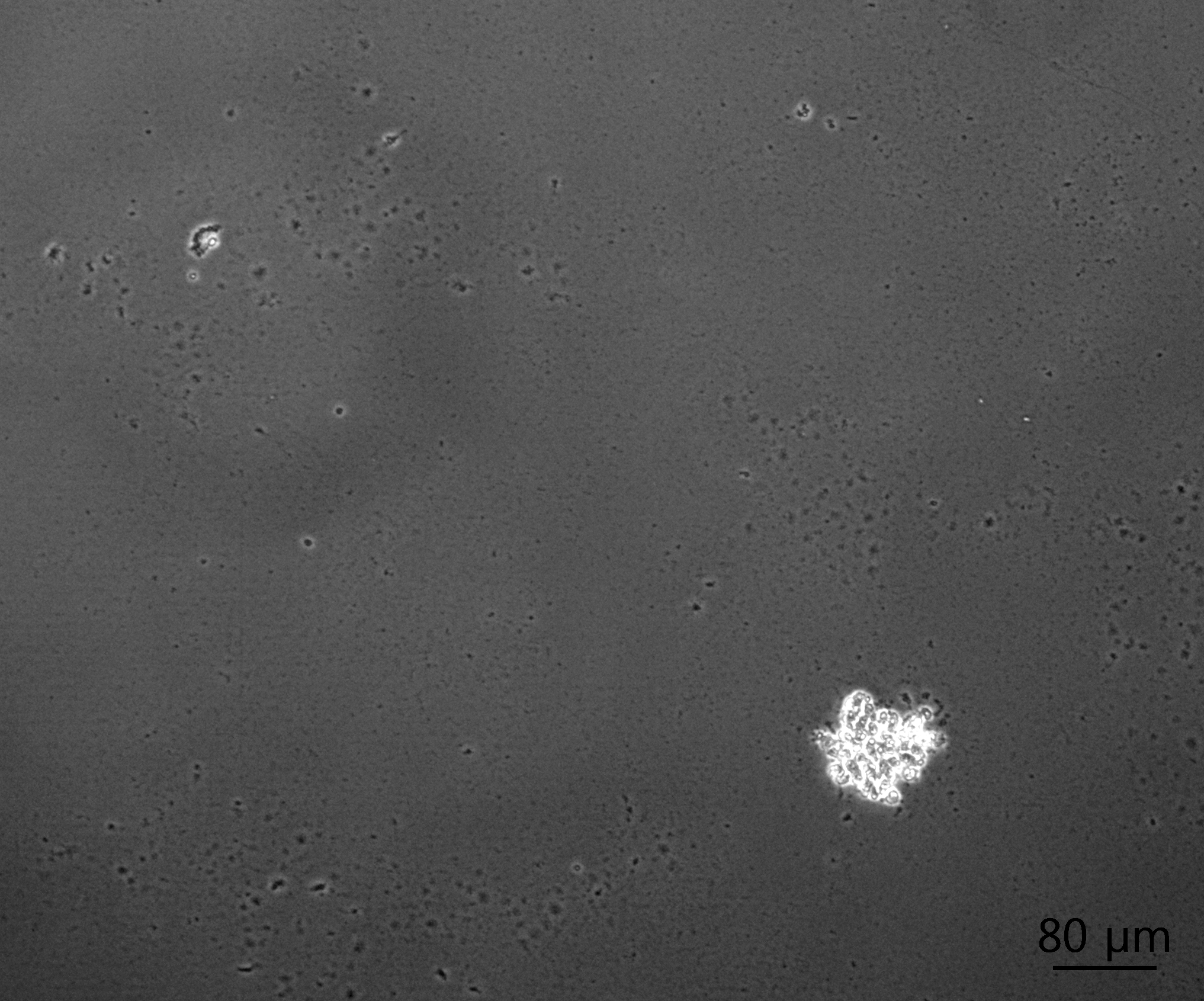

Supplement: Supplementary file 7 — Source data Fig. 5 [file 44319_2025_384_MOESM7_ESM.zip › Figure 5/Fig. 5E/J1 Gys1 KO_No Glc Day3.tif]

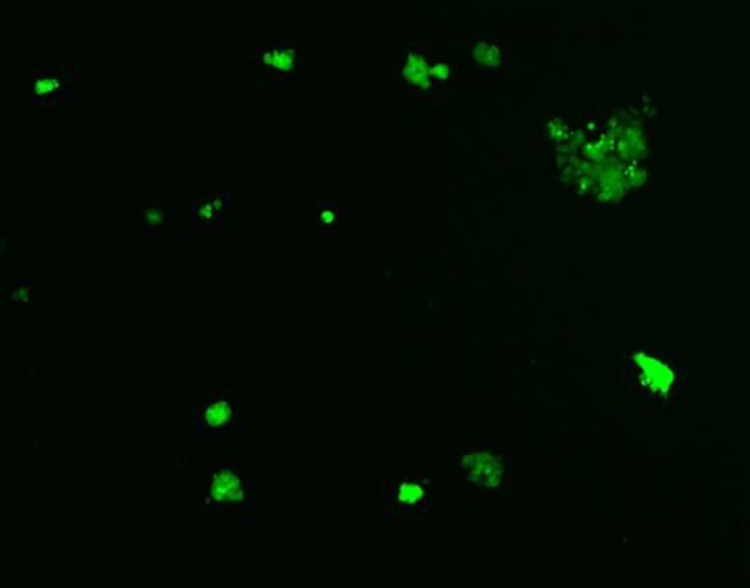

Supplement: Supplementary file 7 — Source data Fig. 5 [file 44319_2025_384_MOESM7_ESM.zip › Figure 5/Fig. 5H/OG-GKO LIF only GFP.tif]

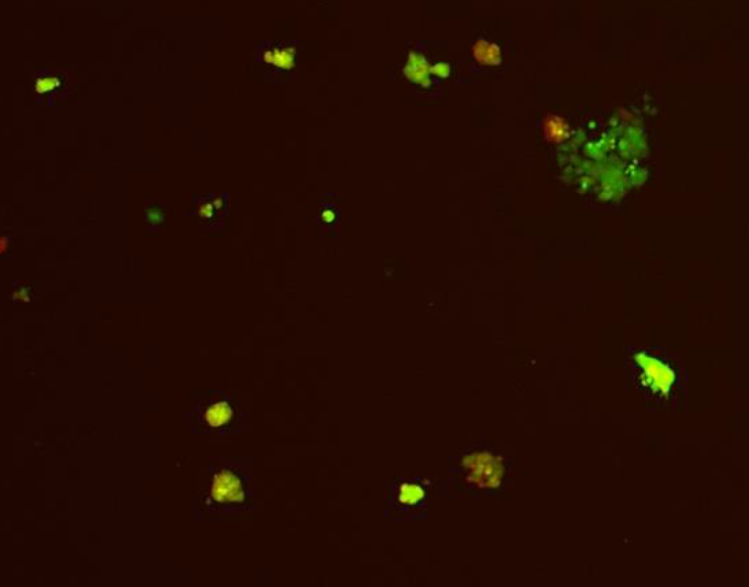

Supplement: Supplementary file 7 — Source data Fig. 5 [file 44319_2025_384_MOESM7_ESM.zip › Figure 5/Fig. 5H/OG-GKO LIF only Merged.tif]

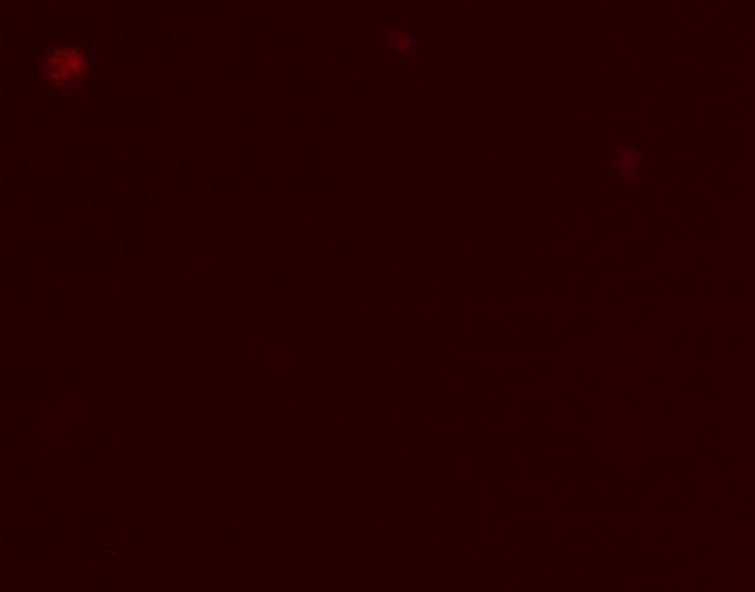

Supplement: Supplementary file 7 — Source data Fig. 5 [file 44319_2025_384_MOESM7_ESM.zip › Figure 5/Fig. 5H/OG-GKO LIF only RFP.tif]

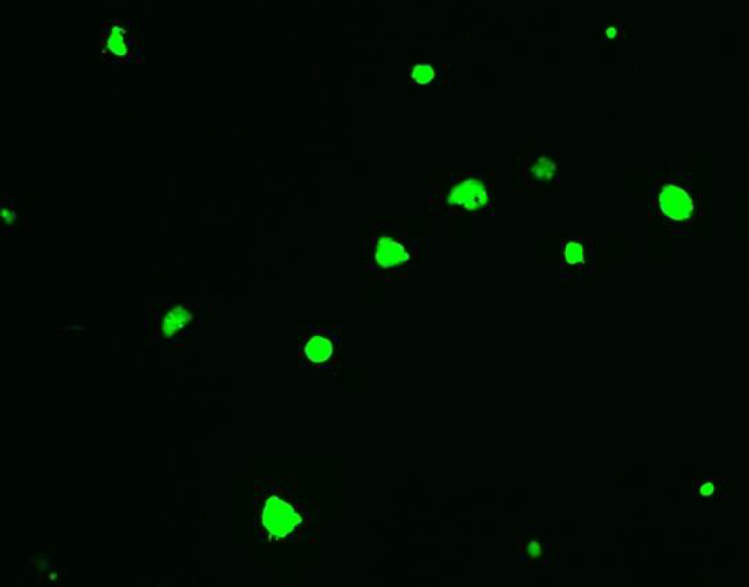

Supplement: Supplementary file 7 — Source data Fig. 5 [file 44319_2025_384_MOESM7_ESM.zip › Figure 5/Fig. 5H/OG-GKO LIF2i GFP.tif]

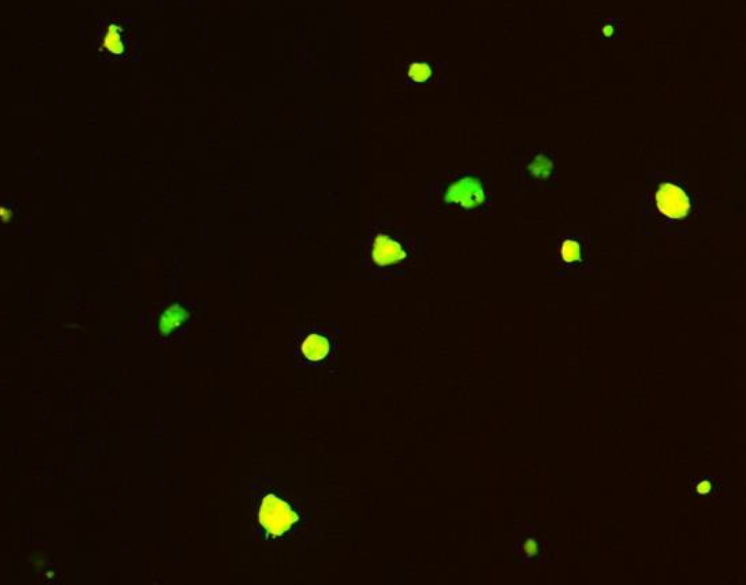

Supplement: Supplementary file 7 — Source data Fig. 5 [file 44319_2025_384_MOESM7_ESM.zip › Figure 5/Fig. 5H/OG-GKO LIF2i Merged.tif]

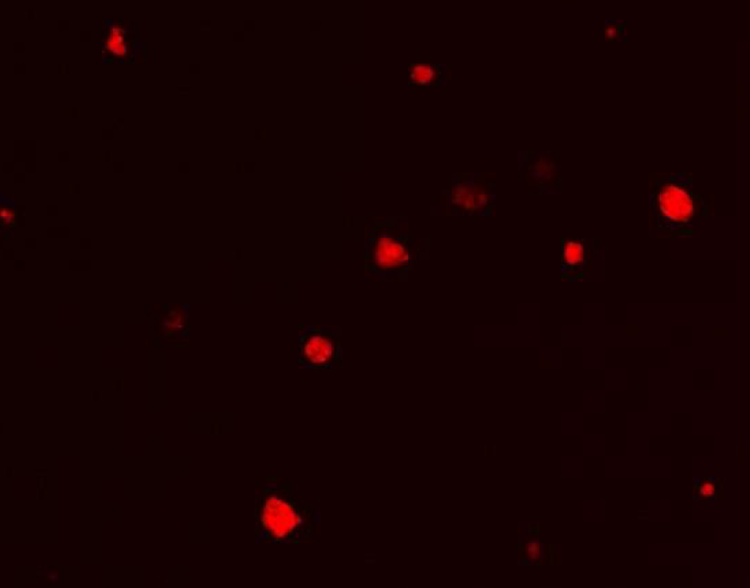

Supplement: Supplementary file 7 — Source data Fig. 5 [file 44319_2025_384_MOESM7_ESM.zip › Figure 5/Fig. 5H/OG-GKO LIF2i RFP.tif]

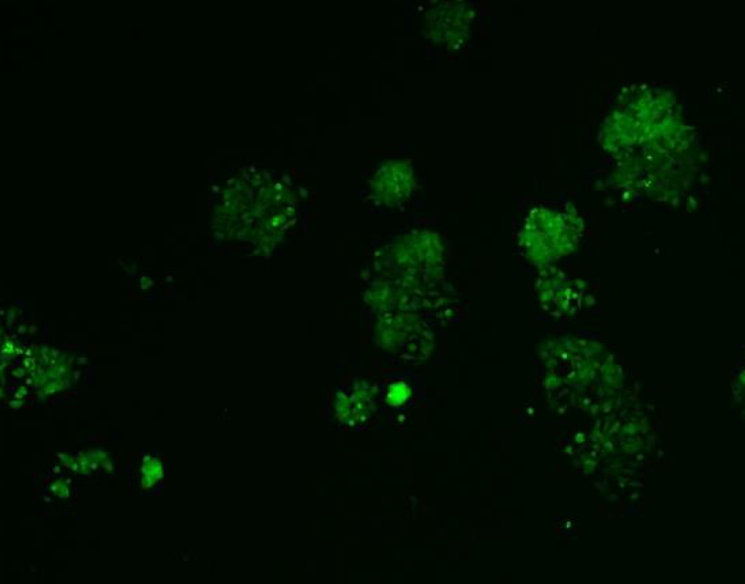

Supplement: Supplementary file 7 — Source data Fig. 5 [file 44319_2025_384_MOESM7_ESM.zip › Figure 5/Fig. 5H/OG-WT LIF only GFP.tif]

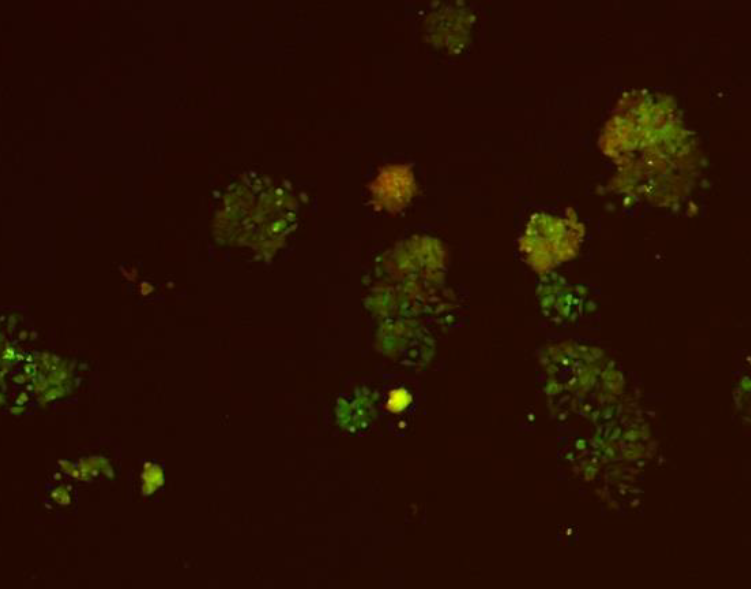

Supplement: Supplementary file 7 — Source data Fig. 5 [file 44319_2025_384_MOESM7_ESM.zip › Figure 5/Fig. 5H/OG-WT LIF only Merged.tif]

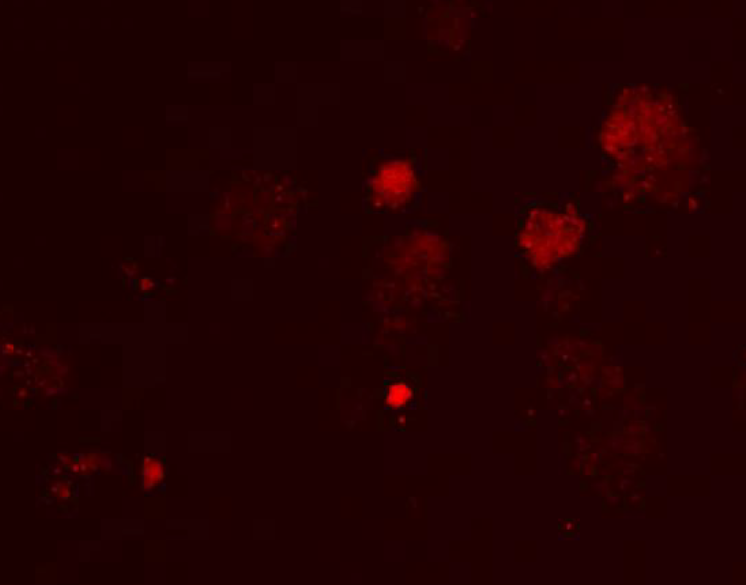

Supplement: Supplementary file 7 — Source data Fig. 5 [file 44319_2025_384_MOESM7_ESM.zip › Figure 5/Fig. 5H/OG-WT LIF only RFP.tif]

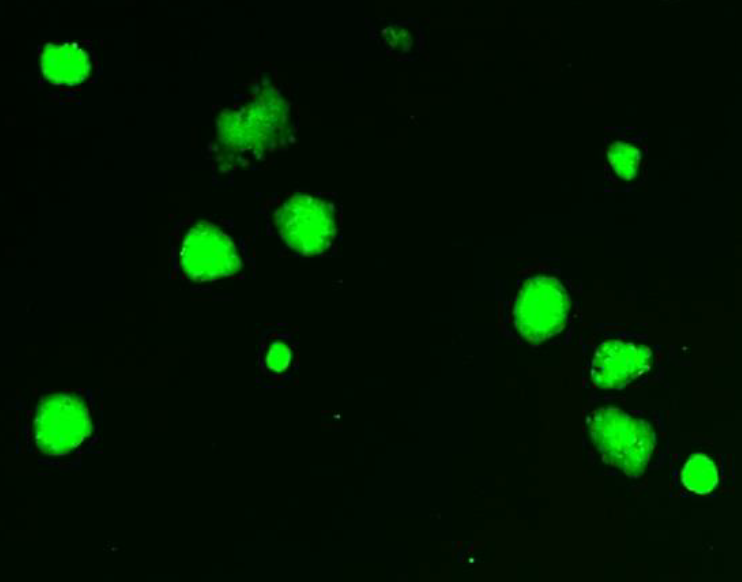

Supplement: Supplementary file 7 — Source data Fig. 5 [file 44319_2025_384_MOESM7_ESM.zip › Figure 5/Fig. 5H/OG-WT LIF2i GFP.tif]

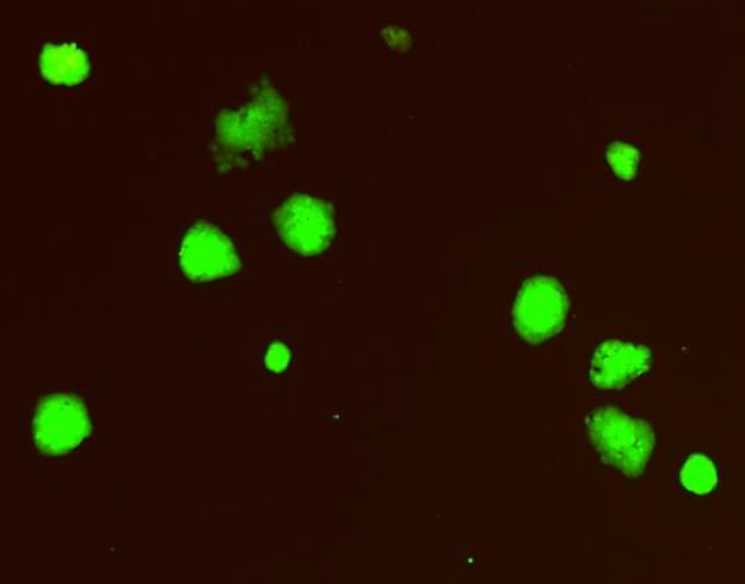

Supplement: Supplementary file 7 — Source data Fig. 5 [file 44319_2025_384_MOESM7_ESM.zip › Figure 5/Fig. 5H/OG-WT LIF2i Merged.tif]

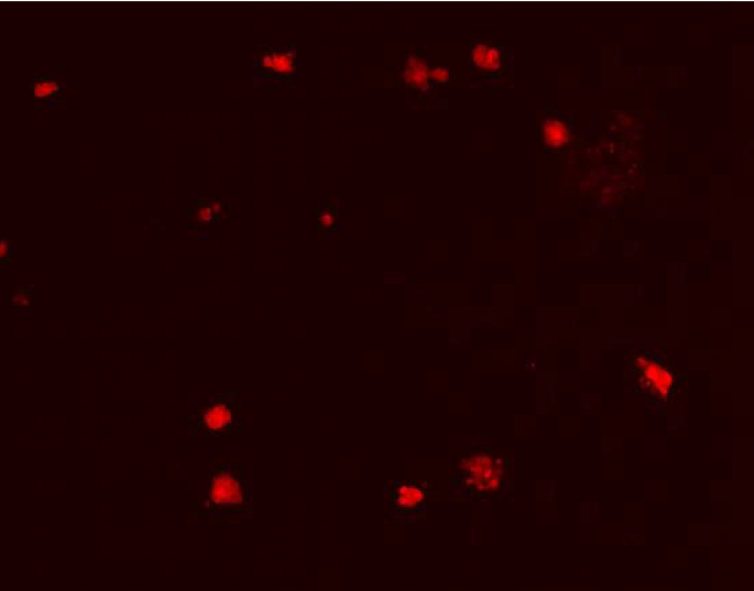

Supplement: Supplementary file 7 — Source data Fig. 5 [file 44319_2025_384_MOESM7_ESM.zip › Figure 5/Fig. 5H/OG-WT LIF2i RFP.tif]

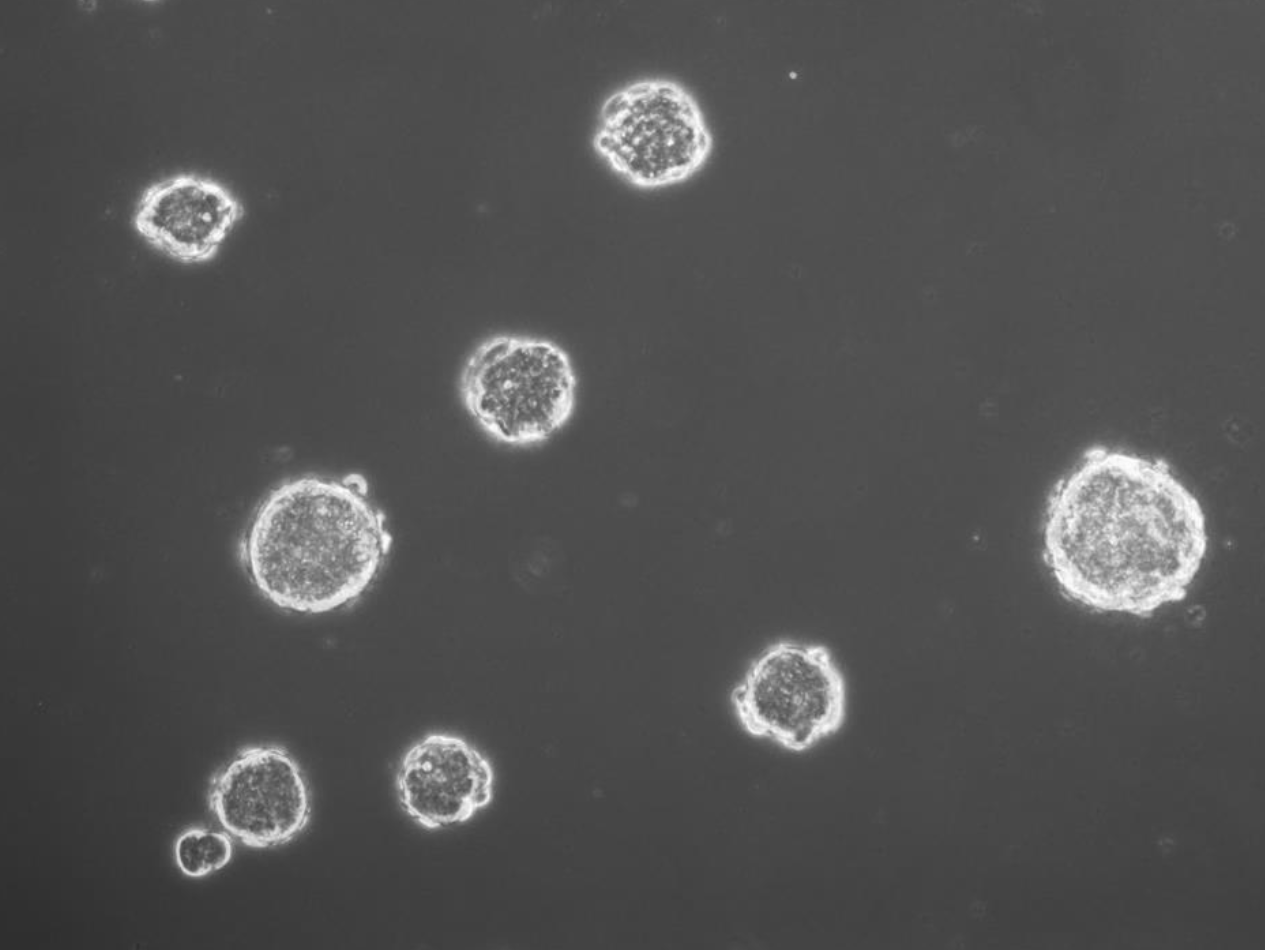

Supplement: Supplementary file 8 — Source data Fig. 6 [file 44319_2025_384_MOESM8_ESM.zip › Figure 6/Fig. 6F/J1 AKO.tif]

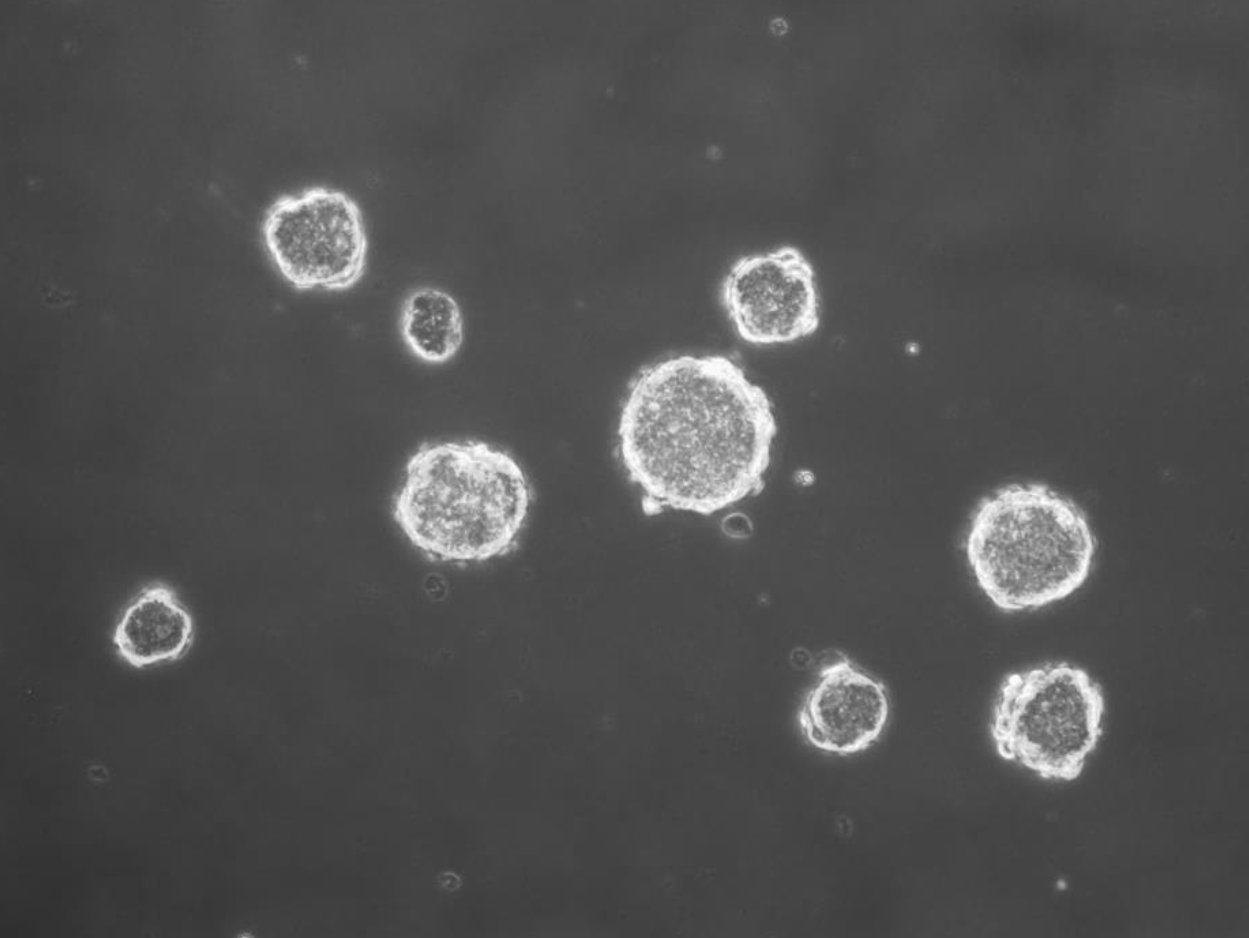

Supplement: Supplementary file 8 — Source data Fig. 6 [file 44319_2025_384_MOESM8_ESM.zip › Figure 6/Fig. 6F/J1 Cont.tif]

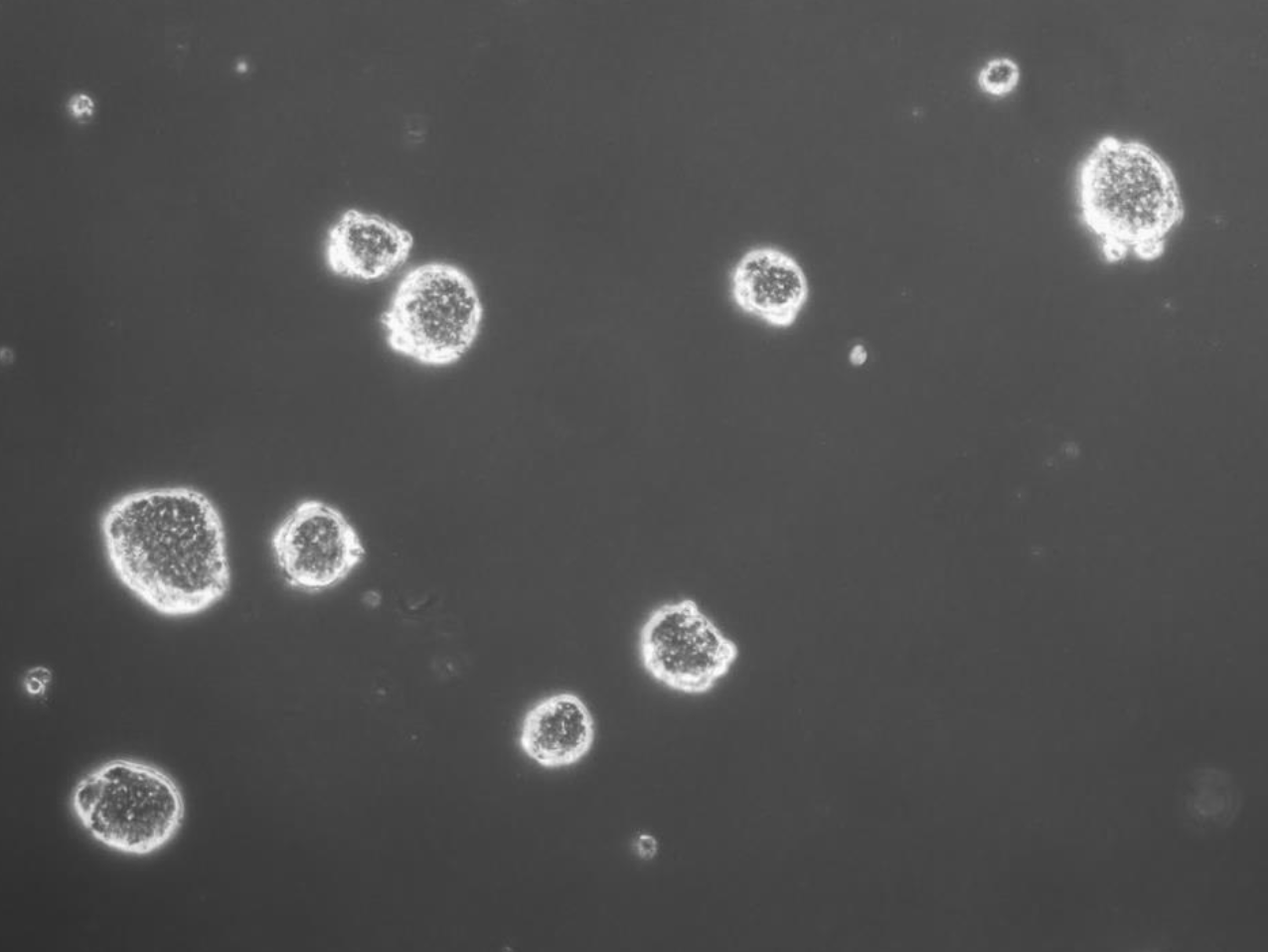

Supplement: Supplementary file 8 — Source data Fig. 6 [file 44319_2025_384_MOESM8_ESM.zip › Figure 6/Fig. 6F/J1 DKO.tif]

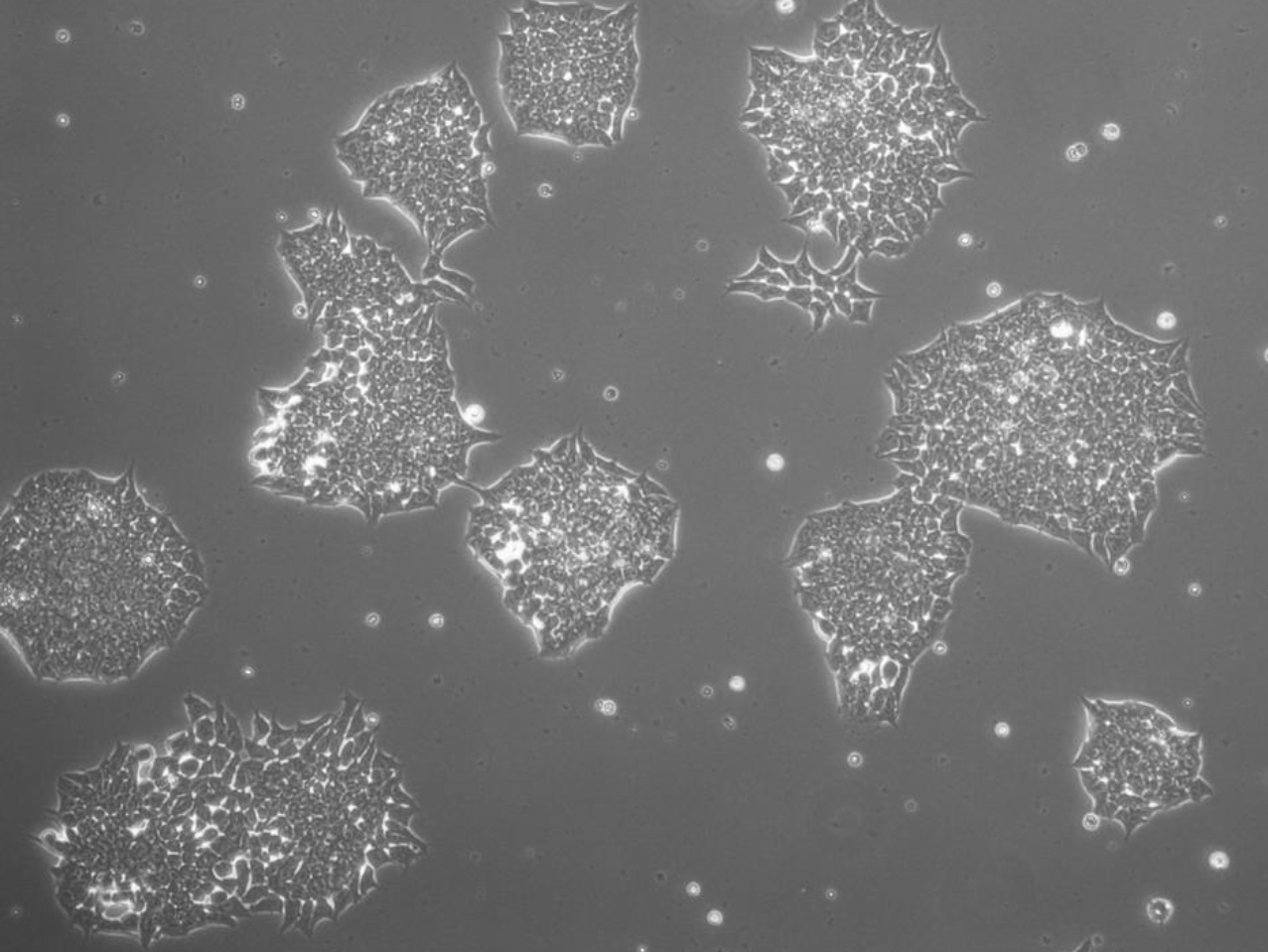

Supplement: Supplementary file 8 — Source data Fig. 6 [file 44319_2025_384_MOESM8_ESM.zip › Figure 6/Fig. 6F/J1 GKO.tif]

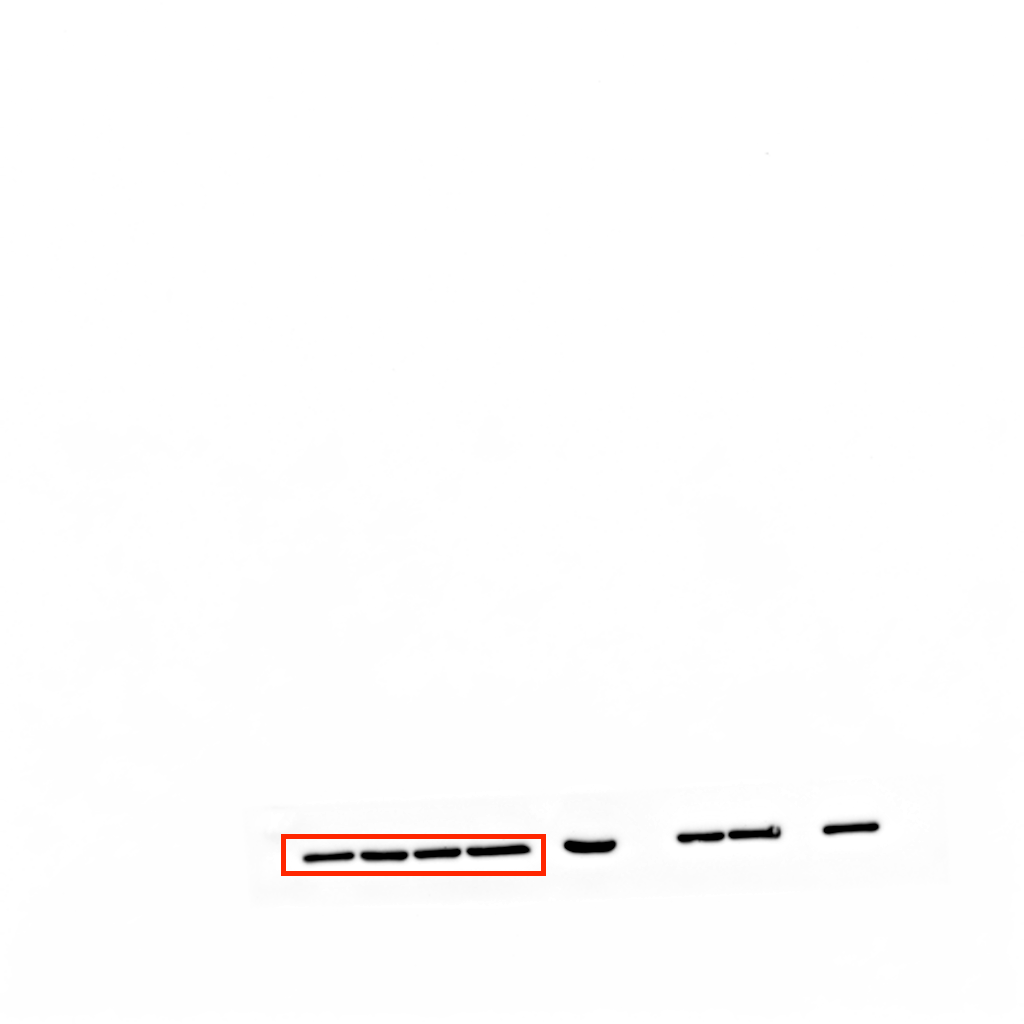

Supplement: Supplementary file 8 — Source data Fig. 6 [file 44319_2025_384_MOESM8_ESM.zip › Figure 6/Fig. 6H/aTUB.TIF]

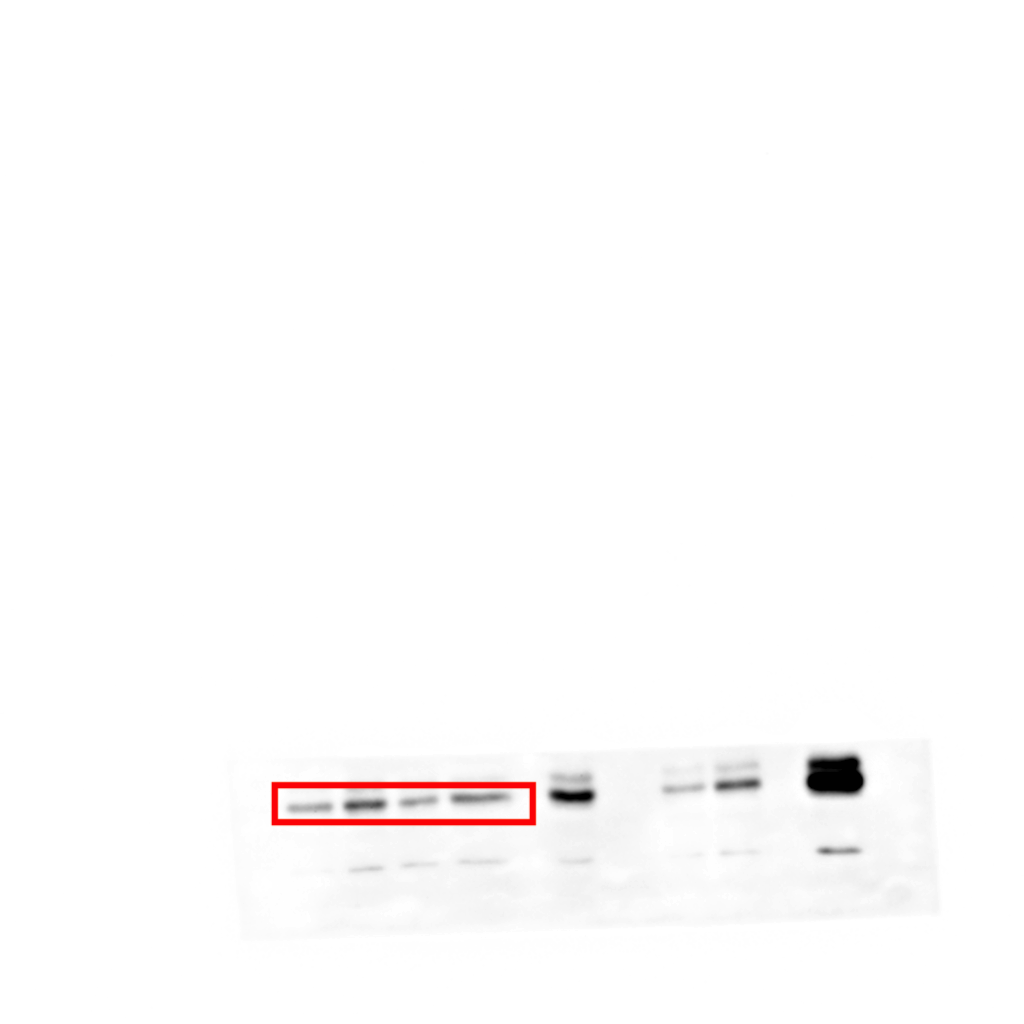

Supplement: Supplementary file 8 — Source data Fig. 6 [file 44319_2025_384_MOESM8_ESM.zip › Figure 6/Fig. 6H/OTX2.TIF]

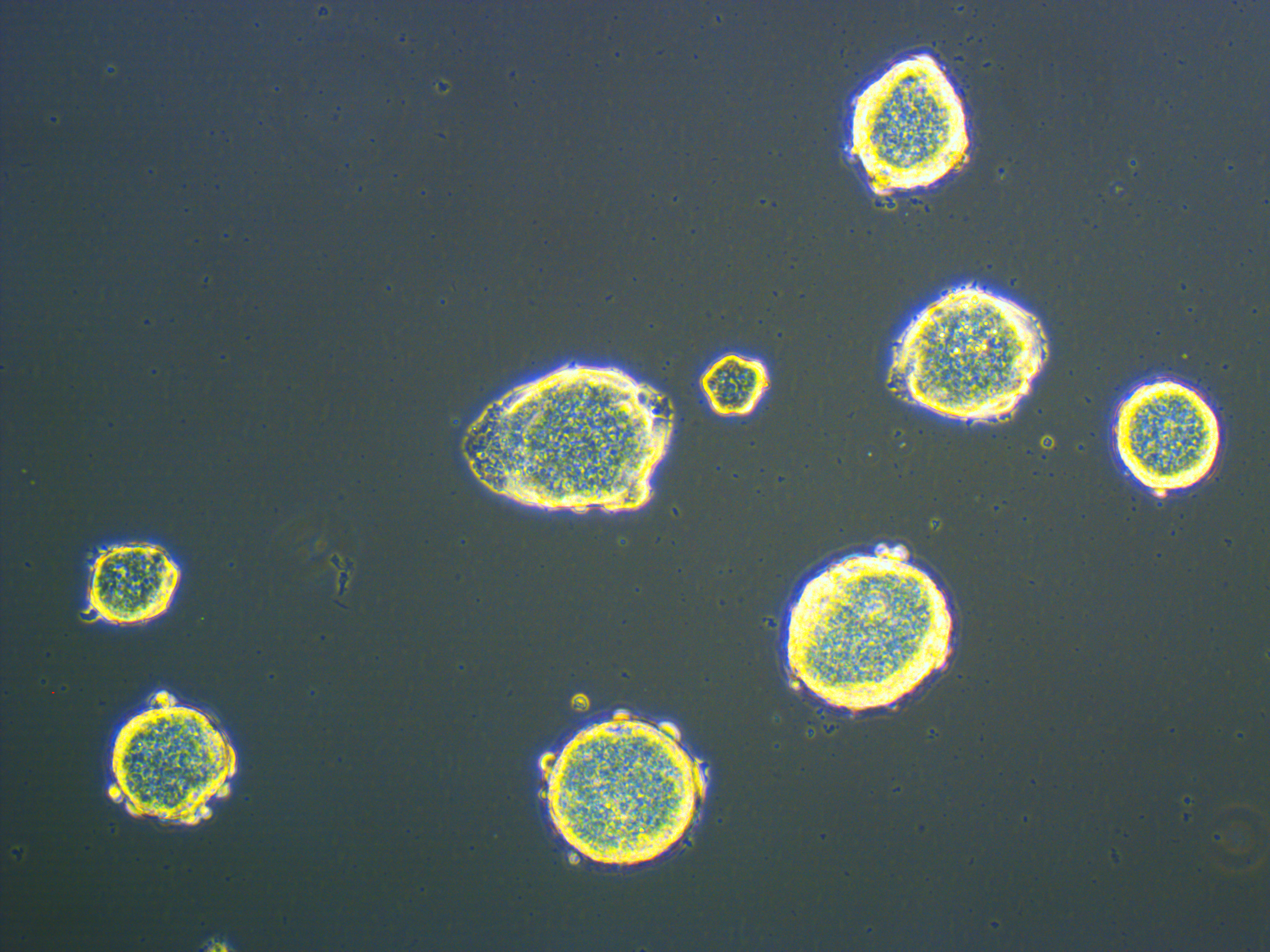

Supplement: Supplementary file 8 — Source data Fig. 6 [file 44319_2025_384_MOESM8_ESM.zip › Figure 6/Fig. 6K/2iL AKO.tif]

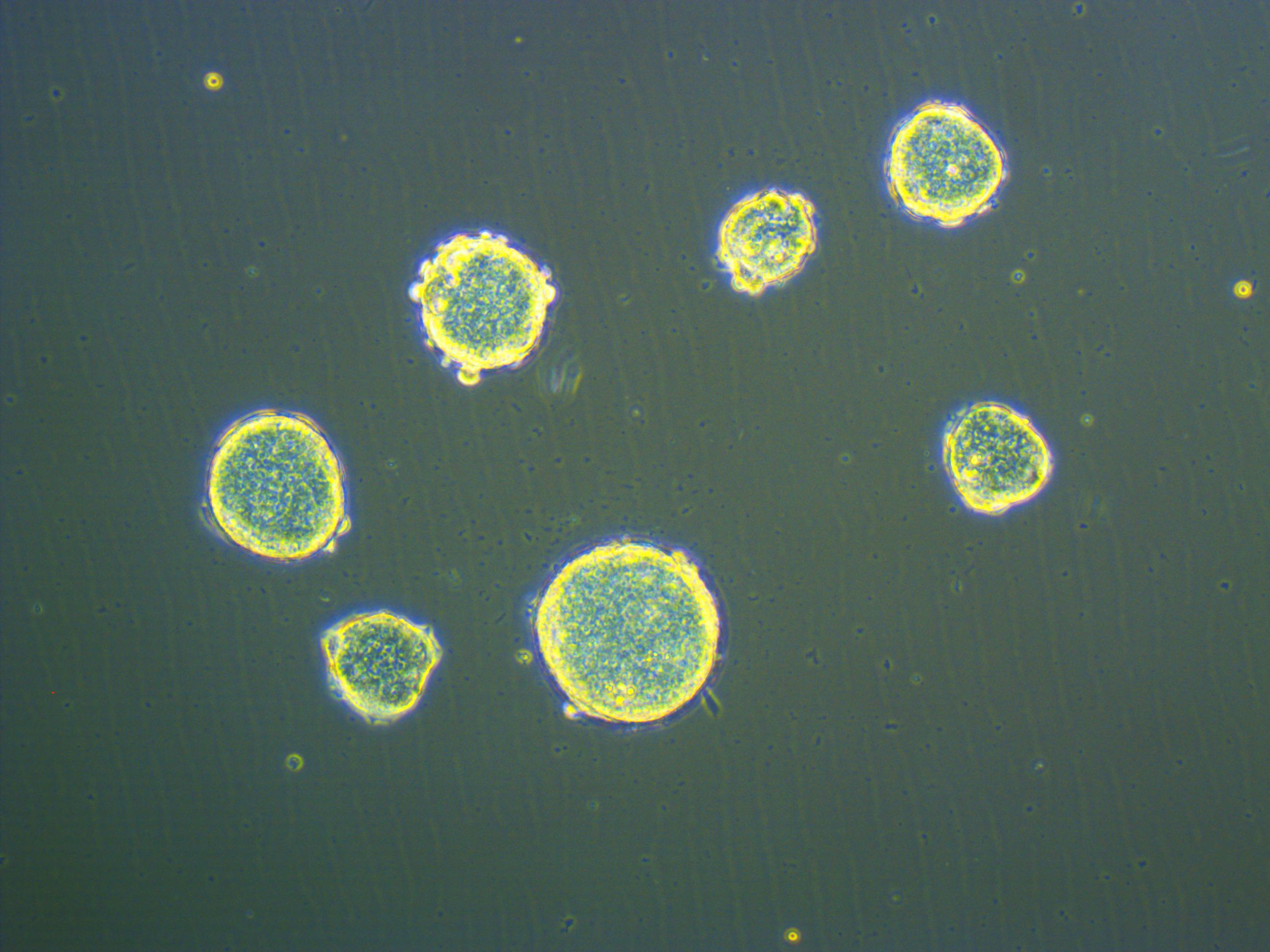

Supplement: Supplementary file 8 — Source data Fig. 6 [file 44319_2025_384_MOESM8_ESM.zip › Figure 6/Fig. 6K/2iL Cont.tif]

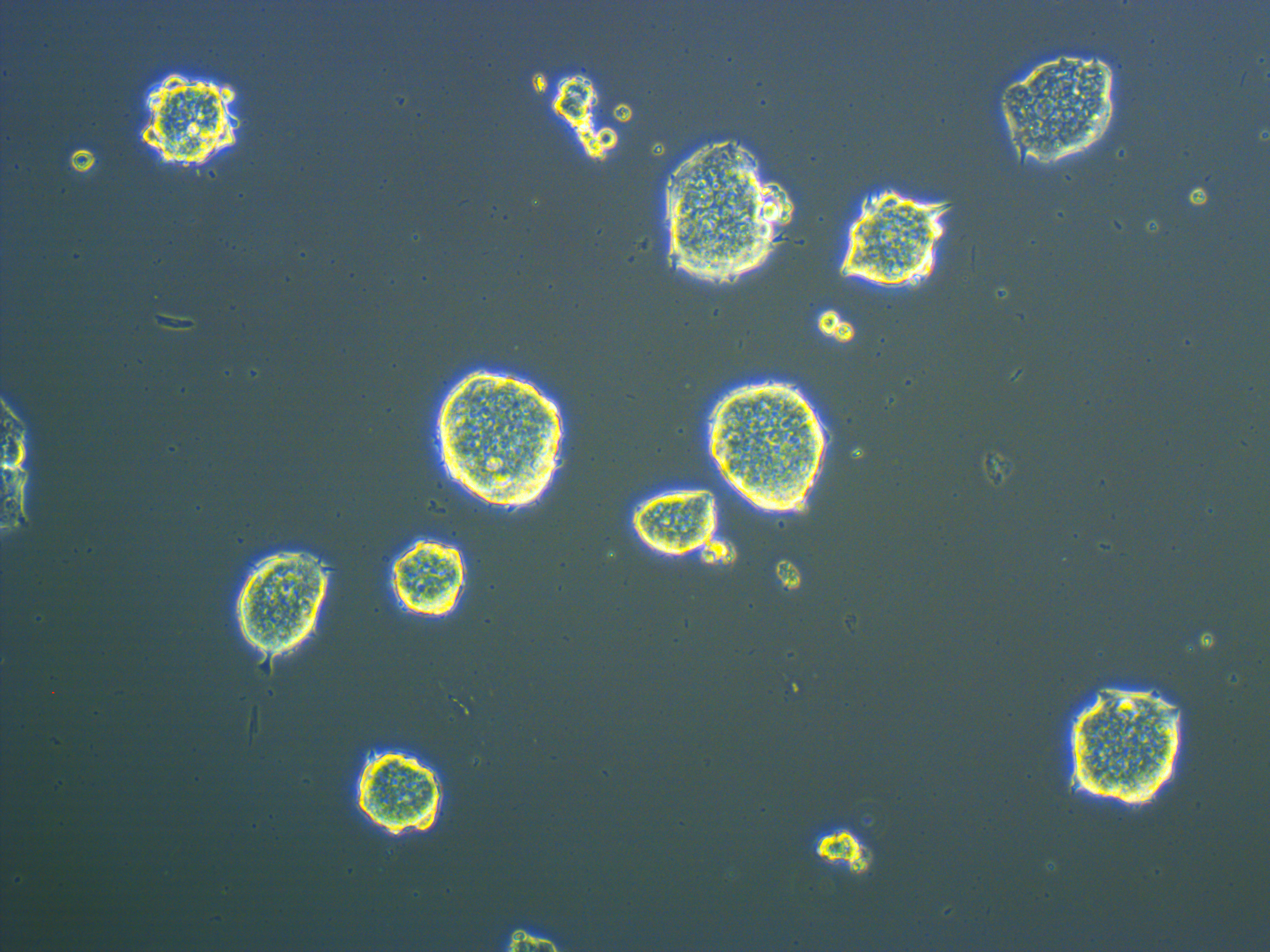

Supplement: Supplementary file 8 — Source data Fig. 6 [file 44319_2025_384_MOESM8_ESM.zip › Figure 6/Fig. 6K/2iL DKO.tif]

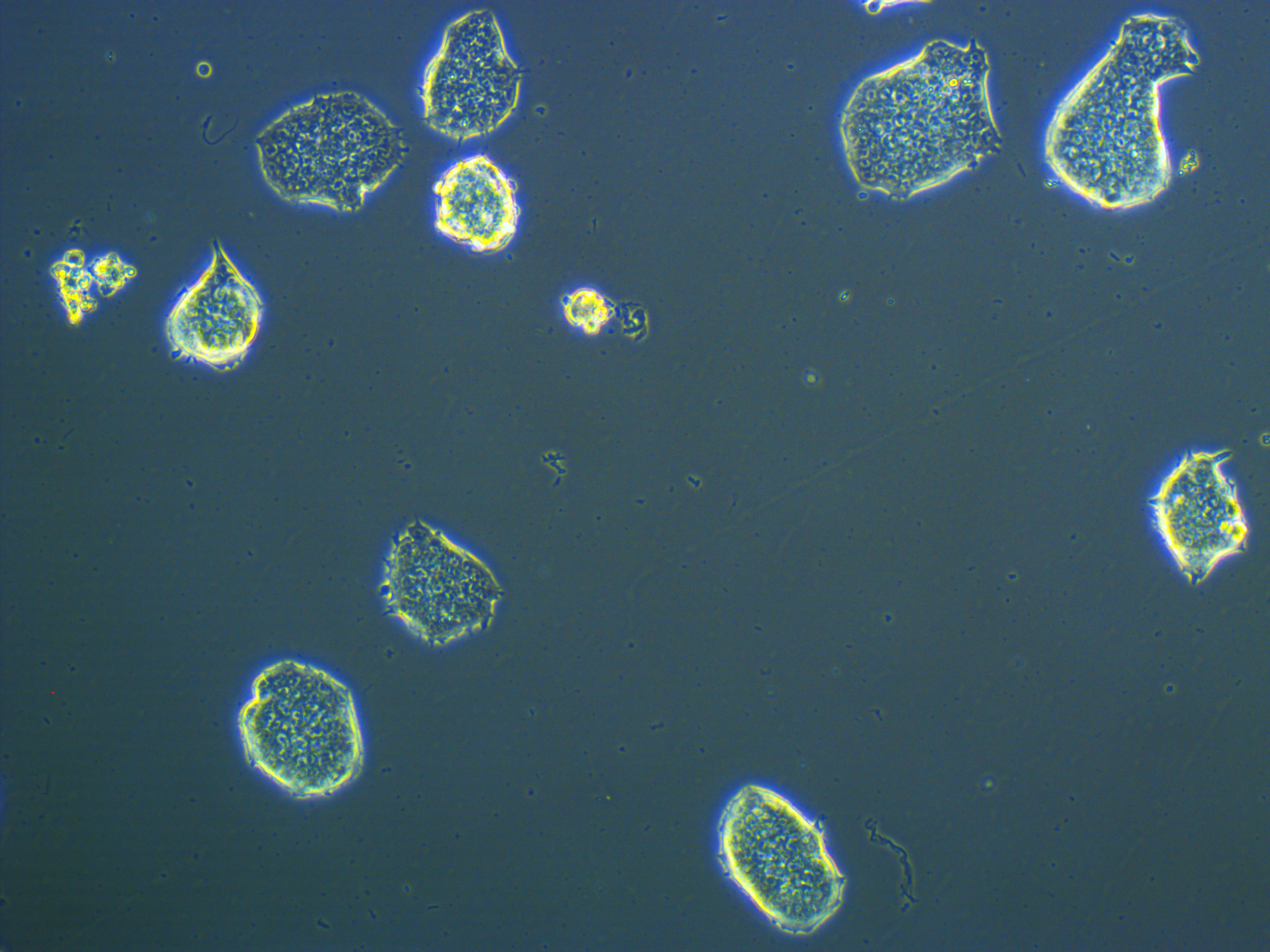

Supplement: Supplementary file 8 — Source data Fig. 6 [file 44319_2025_384_MOESM8_ESM.zip › Figure 6/Fig. 6K/2iL GKO.tif]

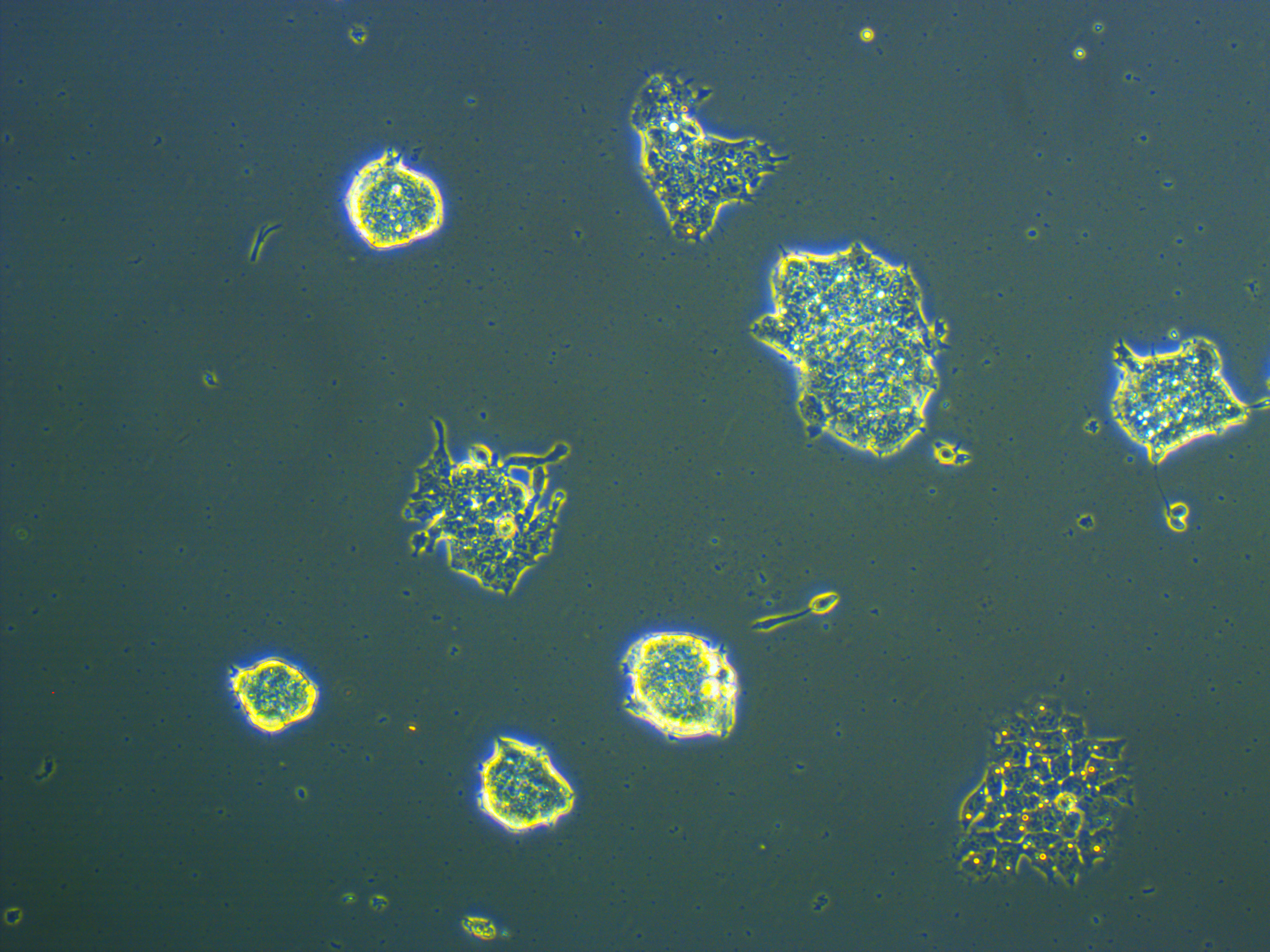

Supplement: Supplementary file 8 — Source data Fig. 6 [file 44319_2025_384_MOESM8_ESM.zip › Figure 6/Fig. 6K/FA AKO.tif]

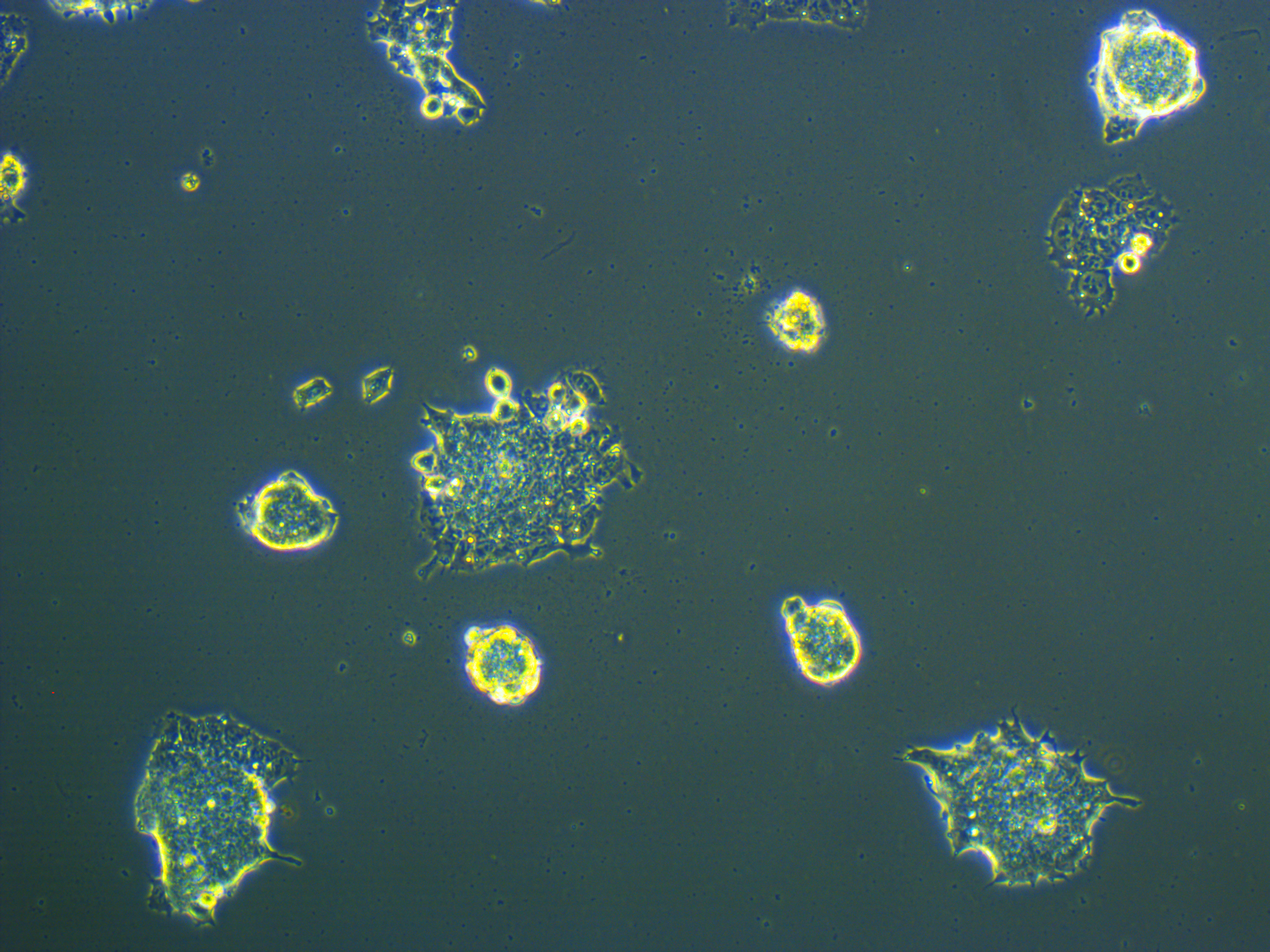

Supplement: Supplementary file 8 — Source data Fig. 6 [file 44319_2025_384_MOESM8_ESM.zip › Figure 6/Fig. 6K/FA Cont.tif]

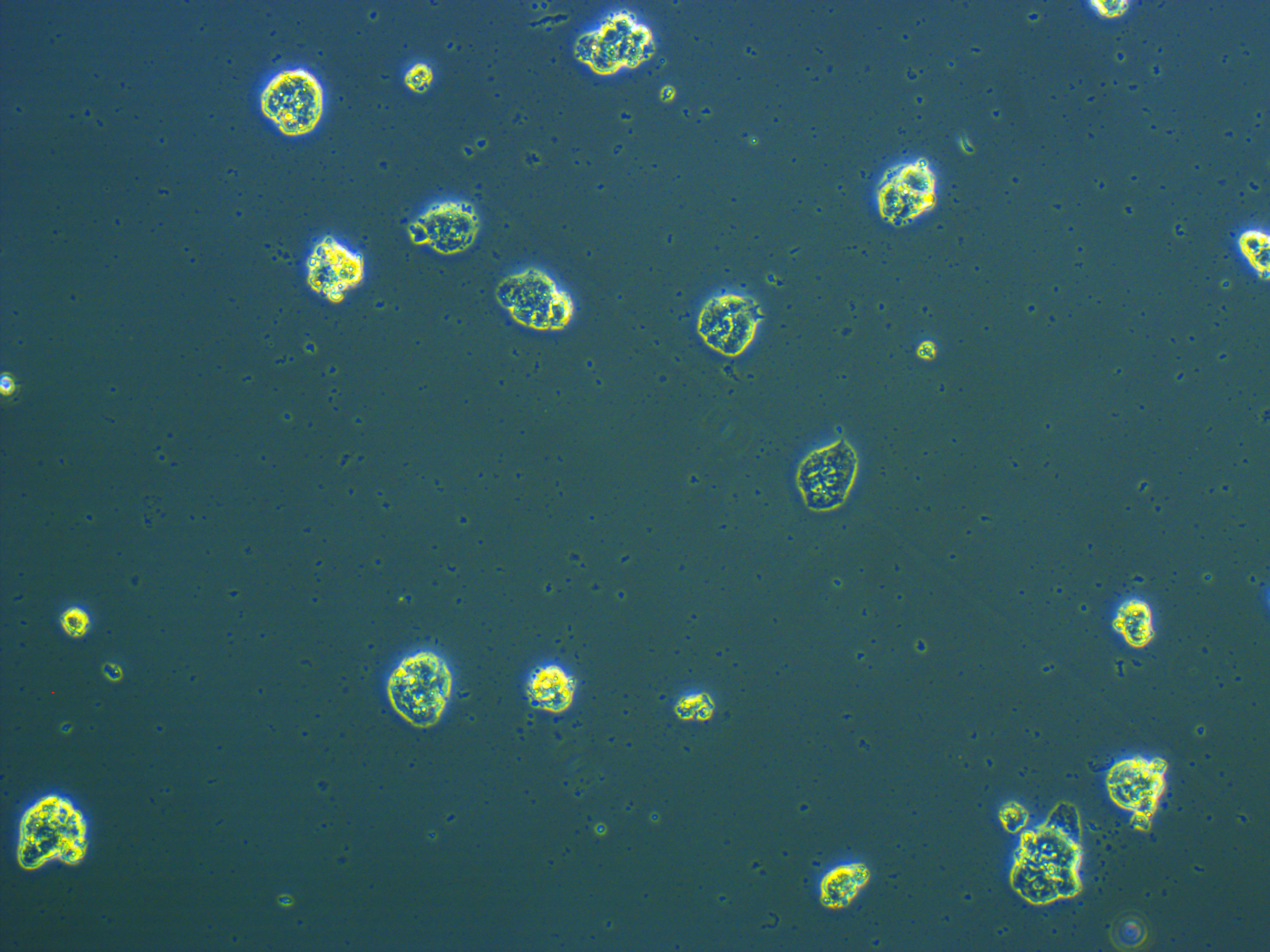

Supplement: Supplementary file 8 — Source data Fig. 6 [file 44319_2025_384_MOESM8_ESM.zip › Figure 6/Fig. 6K/FA DKO.tif]

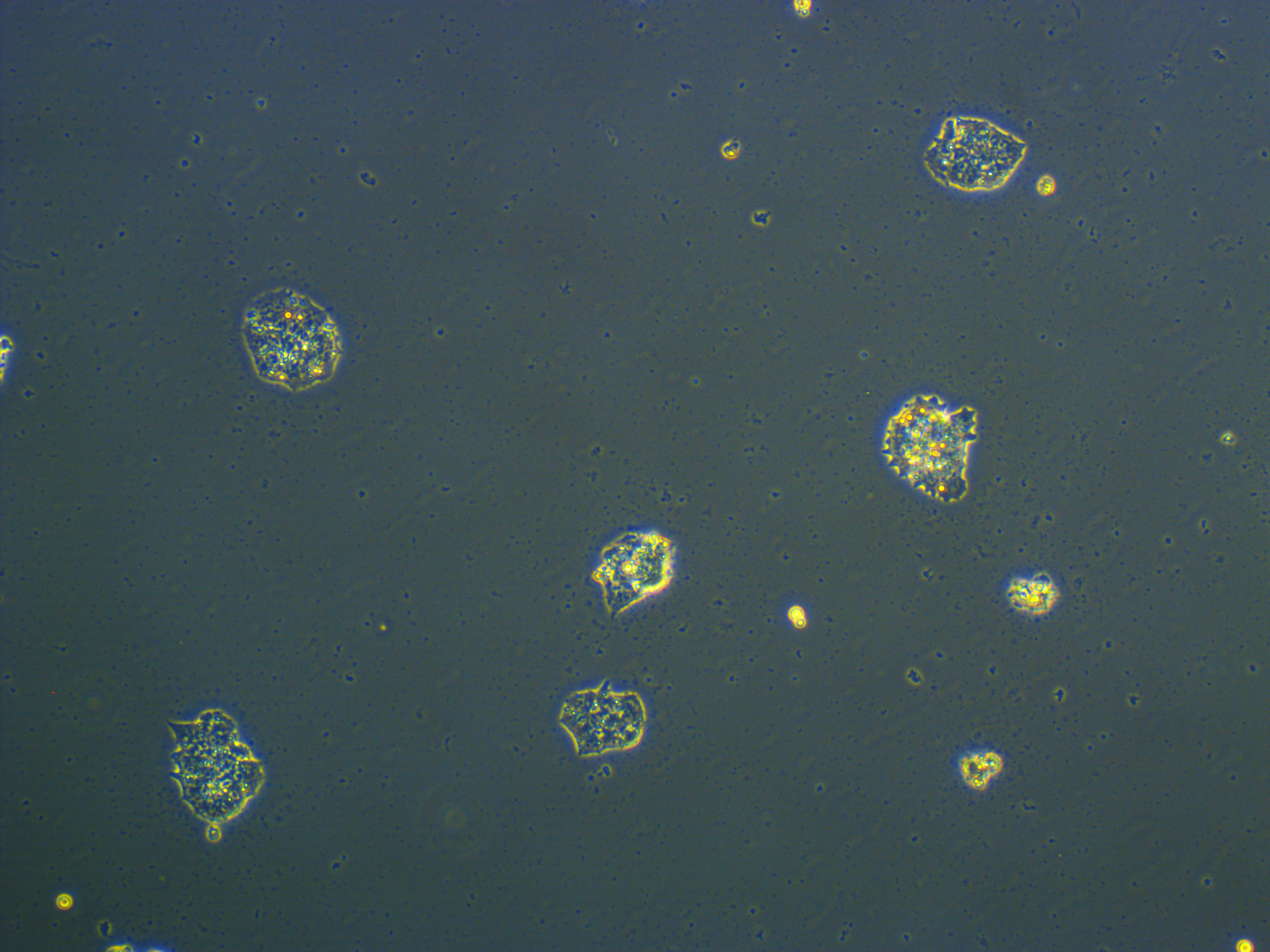

Supplement: Supplementary file 8 — Source data Fig. 6 [file 44319_2025_384_MOESM8_ESM.zip › Figure 6/Fig. 6K/FA GKO.tif]
