# Supplementary material for: AMPK activation by glycogen expenditure primes the exit of naïve pluripotency
Source: EMBO Rep. 2025 Feb 17;26(6):1504–27. doi: 10.1038/s44319-025-00384-x (PMC11933299; doi:10.1038/s44319-025-00384-x)
Supplement: Supplementary file 9 — Expanded View Figures [file 44319_2025_384_MOESM9_ESM.pdf]

Expanded View Figures

Figure EV1. Phosphoproteomic analysis of temporal kinase activity.

(**A**) Volcano plot of differentially phosphorylated proteins after 1, 2 and 6 h of treatment ($n = 4$) compared to SL ($n = 8$). We employed moderated t-test which were implemented in the R package limma (v3.62.1). Differentially phosphorylated proteins were selected based on a Benjamini–Hochberg-adjusted P-value less than 0.05 and an absolute fold-change greater than 0 as the cut-offs. (**B**) Phosphoproteins phosphorylated by MAPK1, ERK1 and GSk3 among the phosphoproteins belonging to cluster 3. (**C**) PCA of phosphoproteome data from 2i treatment (0.5-, 1-, 2-, and 6-h samples displayed in red gradient color) and Cdk8i treatment (0.5, 1, 2, and 6-h samples displayed in green gradient color). (**D**) Hierarchical clustering results representing four phosphoproteins' clusters showing distinct phosphoproteome changes at various time points after CDK8i treatment. The top five kinase categories significantly enriched in each phosphoprotein cluster. (**E**) Phosphoproteins phosphorylated by AMPK among the phosphoproteins belonging to cluster 1.

 © The Author(s)

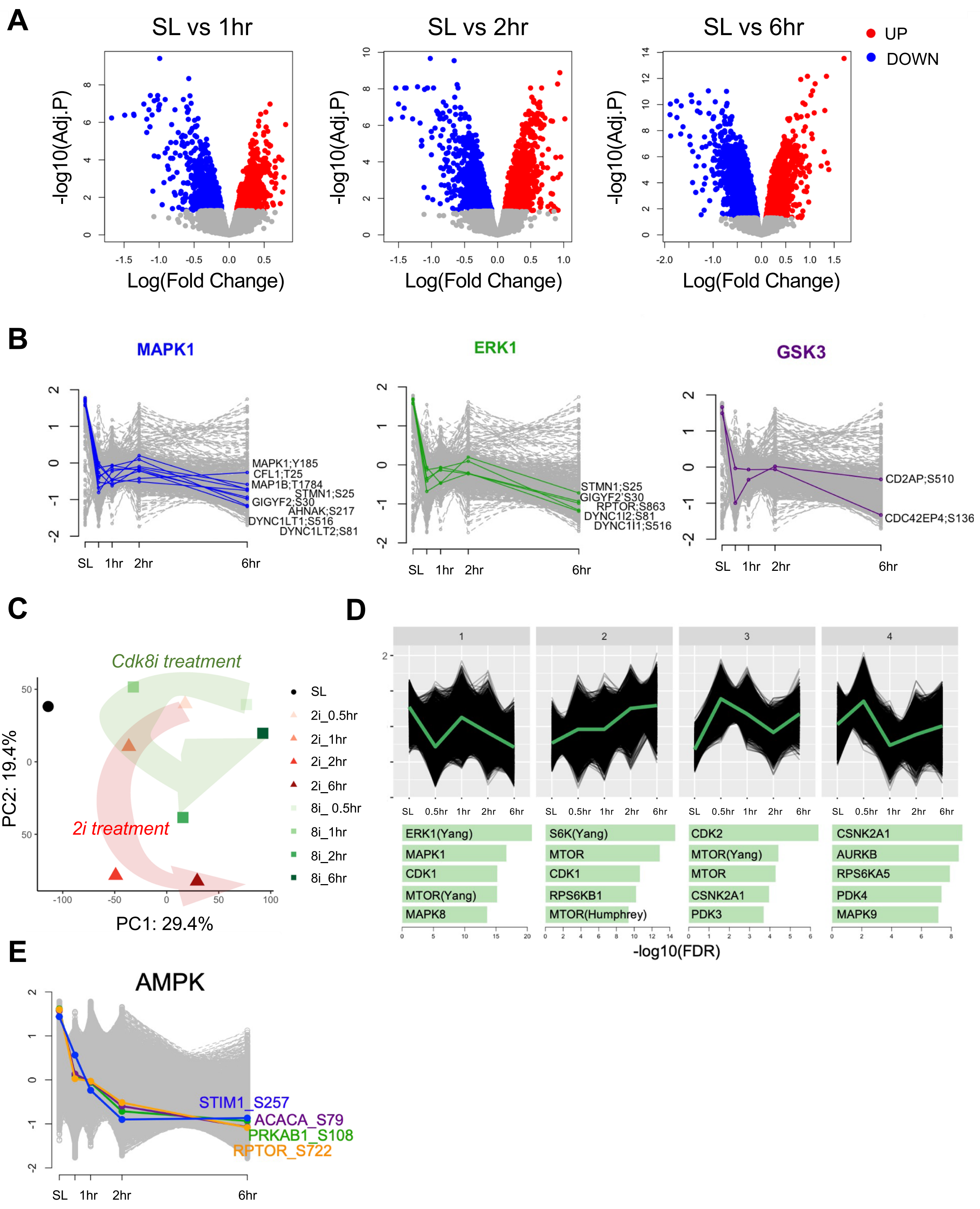
A
SL vs 1hr
SL vs 2hr
SL vs 6hr
UP
DOWN
-log10(Adj.P)
Log(Fold Change)
B
MAPK1
ERK1
GSK3
MAPK1;Y185
CFL1;T25
MAP1B;T1784
STMN1;S25
GIGYF2;S30
AHNAK;S217
DYNC1LT1;S516
DYNC1LT2;S81
STMN1;S25
GIGYF2;S30
RPTOR;S863
DYNC1I2;S81
DYNC1I1;S516
CD2AP;S510
CDC42EP4;S136
SL 1hr 2hr 6hr
C
Cdk8i treatment
2i treatment
PC1: 29.4%
PC2: 19.4%
SL
2i_0.5hr
2i_1hr
2i_2hr
2i_6hr
8i_0.5hr
8i_1hr
8i_2hr
8i_6hr
D
SL 0.5hr 1hr 2hr 6hr
ERK1(Yang)
MAPK1
CDK1
MTOR(Yang)
MAPK8
S6K(Yang)
MTOR
CDK1
RPS6KB1
MTOR(Humphrey)
CDK2
MTOR(Yang)
MTOR
CSNK2A1
PDK3
CSNK2A1
AURKB
RPS6KA5
PDK4
MAPK9
-log10(FDR)
E
AMPK
STIM1_S257
ACACA_S79
PRKAB1_S108
RPTOR_S722

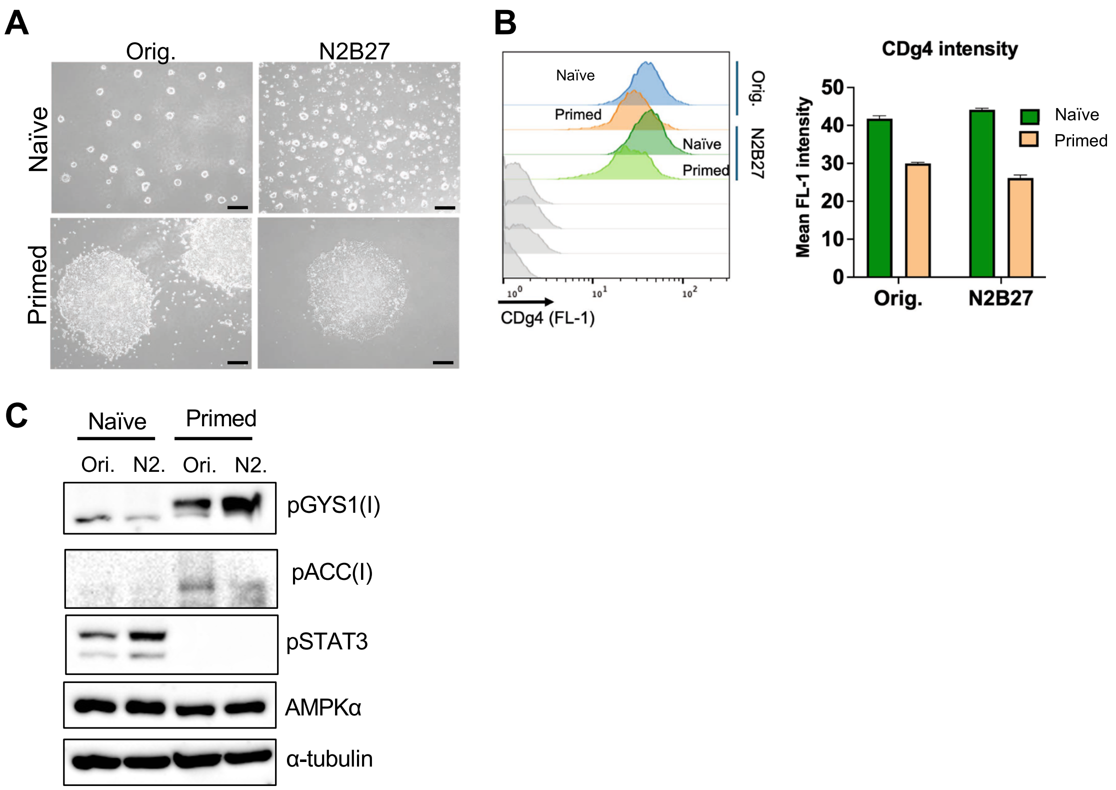

Figure EV2. Higher glycogen amount and increased GYS1 activity in Naïve ESCs.

(**A**) Phase contrast images of naïve (J1) and primed (PJ1) mESCs cultured under DMEM-based media (Org.) or chemically defined N2B27-based media (N2B27). Scale bar = 100 μm. (**B**) Flow cytometric analysis CDg4 with either Org. or N2B27-cultured naïve (J1) and primed (PJ1) mESCs. Quantified mean intensity of CDg4 is shown in right-side graph. (**C**) Immunoblotting analysis of naïve (J1) and primed (PJ1) mESCs cultured with either Org. or N2B27-mediated defined media (N2.). Indicative protein expression of pGYS1, pACC, pSTAT3 and AMPKα is shown, and α-tubulin was used for loading control. Active or inactive phosphorylation of each protein is marked as [A] or [I], respectively.

 © The Author(s)

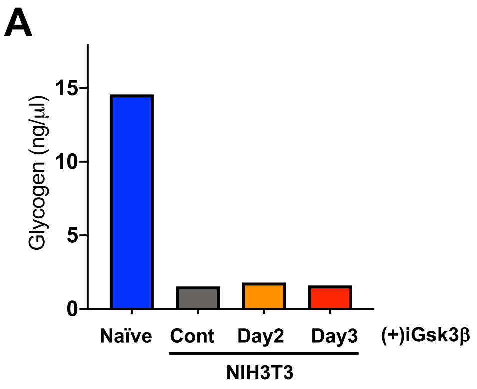

Figure EV3. High glycogen amount in Naïve ESCs, Establishment of AMPK KO cell line.

(**A**) Glycogen amount of naïve (J1) and NIH3T3 cells treated with iGSK3β for 3 days are quantified. (**B**) Immunoblotting analysis from Cont (J1) and Ampk KO J1 mESCs (AKO) with Ampk expression is shown, β-actin is used for internal loading control.

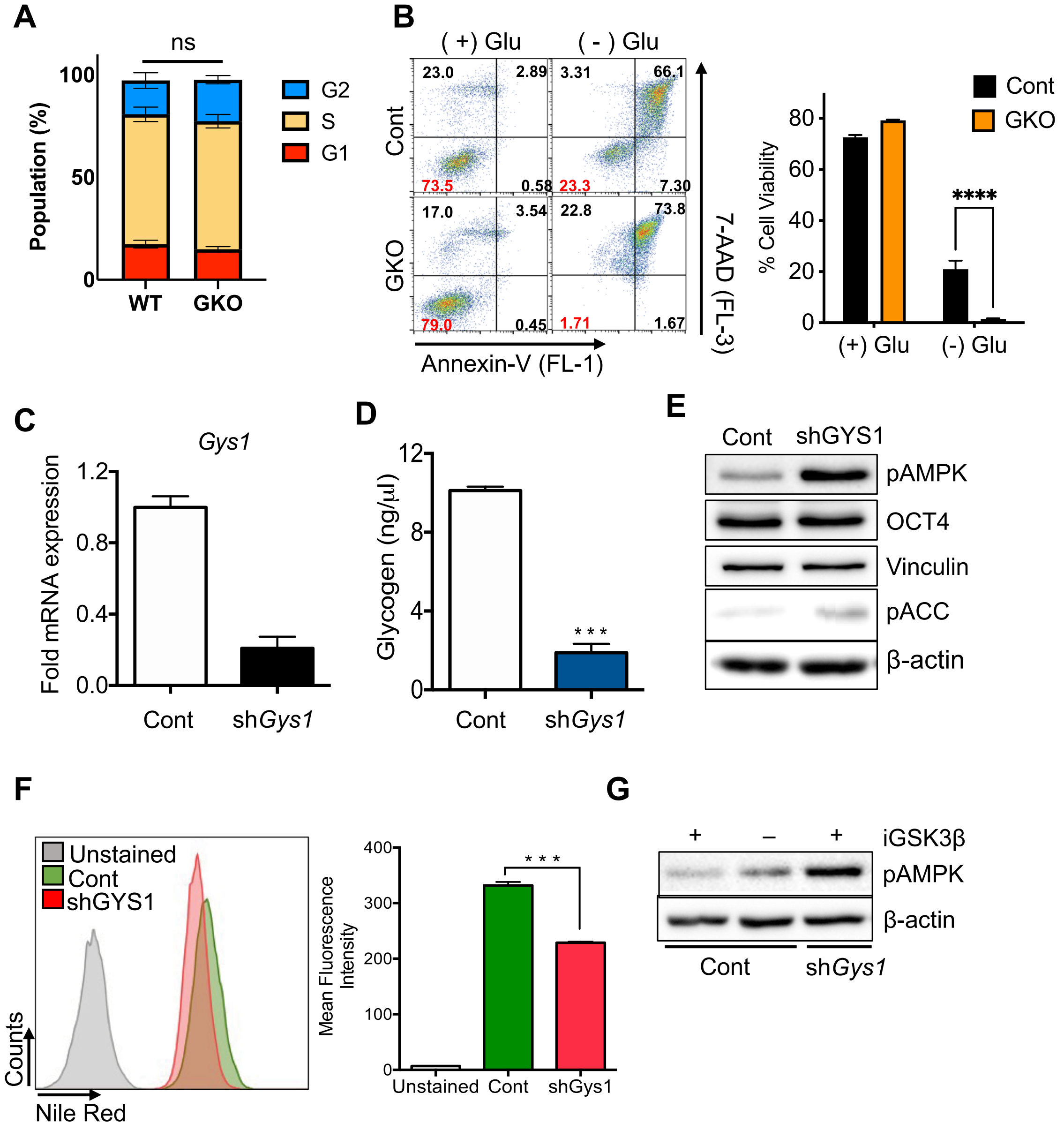

Figure EV4. Establishment of Knockout (GKO) and Knockdown (sh*Gys1*) of *Gys1*.

(**A**) Cell cycle profile analysis with WT (J1) and *Gys1* KO (GKO) mESCs after treatment of Propidium iodide (PI)-staining is quantified. Based on DNA content estimation, each cell cycle stage (G1, S and G2) is analyzed. two-way ANOVA, multiple comparisons, ns indicates statistically not significant ($N = 3$, $n = 3$). (**B**) Flow cytometry for Annexin-V and 7-AAD staining of Cont and GKO with [+Glu] or without [-Glu] Glucose (left panel), quantification of live cell population (rignt panel). 2-way ANOVA, multiple comparisons, (****$P < 0.0001$, $N = 3$, $n = 3$). (**C**) Fold mRNA expression of *Gys1* in before [Cont] and after transient knock down of *Gys1* [sh*Gys1*]. (**D**) Intracellular glycogen level before [Cont] and after transient knock down of *Gys1* [sh*Gys1*]. Multiple t-tests (***$P = 0.0003$, $N = 3$, $n = 3$). (**E**) Immunoblotting analysis for indicative proteins (pAmpkα, Oct4, Vinculin, pAcc, β-actin was used for loading control) before [Cont] and after transient knock down of *Gys1* [sh*Gys1*]. (**F**) Flow Cytometry of Nile Red staining before [Cont] and after transient knock down of *Gys1* [sh*Gys1*] (left panel), quantification of Mean Fluorescence Intensity (right panel). Multiple t-tests (***$P = 0.0002$, $N = 3$, $n = 3$). (**G**) Immunoblotting analysis for pAmpkα (β-actin was used for loading control) before [Cont] and after transient knock down of *Gys1* [sh*Gys1*]. See also Fig. 3B.

 © The Author(s)

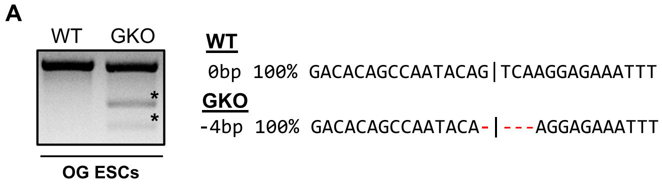

Figure EV5. Establishment of *Gys1*KO (GKO) cell line.

(**A**) T7E1 assay for WT and GKO in OG2$^{+/-}$GOF6$^{+/-}$ cell line (left panel), sequence information of targeted *Gys1* from WT and GKO in OG2$^{+/-}$GOF6$^{+/-}$ cell line (right panel).

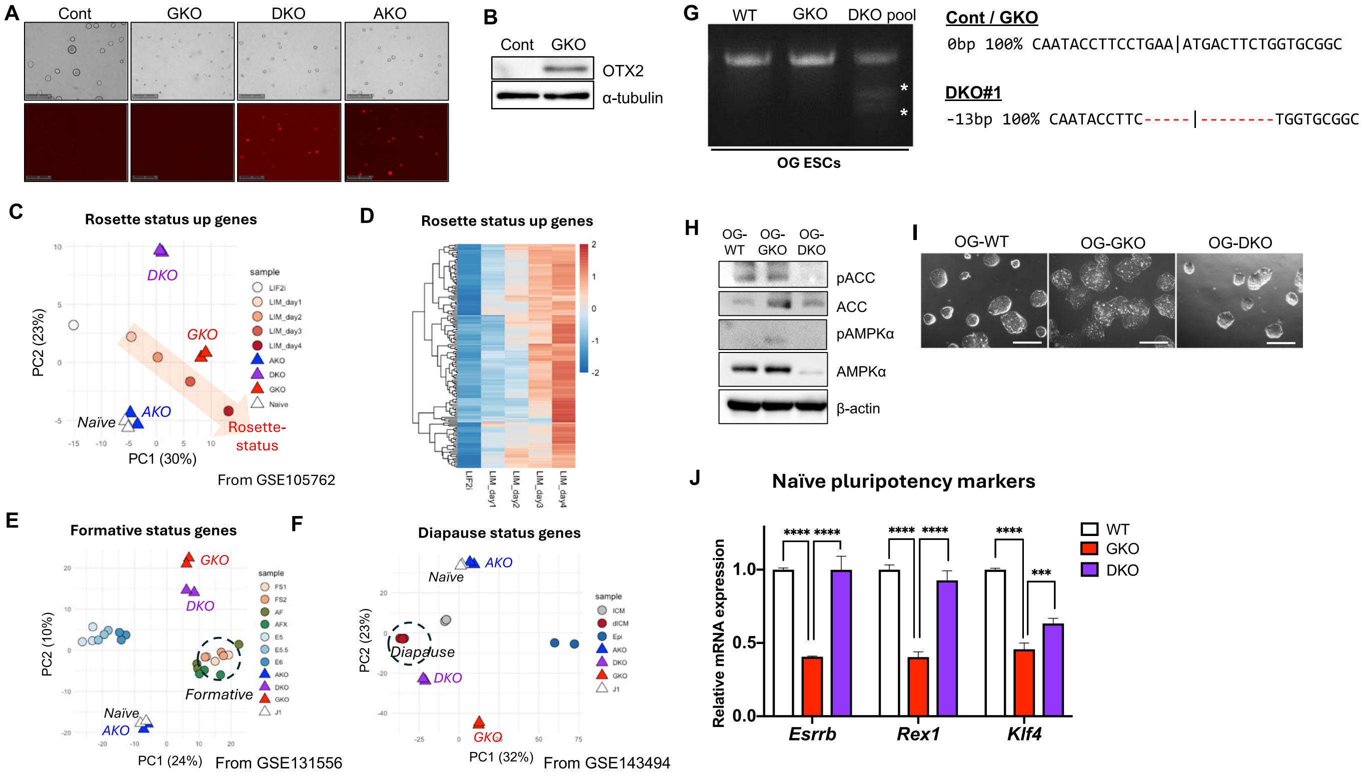

Figure EV6. Establishment of *Gys1* KO (GKO) and *Prkaa1* KO (AKO).

(**A**) Brightfield and RFP images for Cont, GKO, DKO and AKO (scale bar = 500 μm). (**B**) Immunoblotting analysis for indicative proteins (OTX2 in Cont and GKO, α-tubulin was used as a loading control). (**C**) PCA of RNA-seq data from this study (triangle shape; White: Naive (J1), Blue: AKO, Purple: DKO and Red: GKO) and previously published studies (circle shape; Red: LIM medium, Blue: LFAI medium). Samples cultured in the medium containing LIF, PD325901, and the Wnt inhibitor IWP2 were labeled as LIM, with the duration of culture indicated numerically. Samples cultured in the medium containing LIF, FGF2, Activin A, and IWP2 were labeled as LFAI, similarly annotated with the duration of culture. Red arrows indicate changes observed in LIM medium under LIF+2i (PD325901, CHIR99021) conditions, while blue arrows indicate changes observed in LFAI medium under LIF+2i conditions. (**D**) Gene set variation analysis (GSVA) of Naive and primed pluripotency signature with LIF2i, LIM day1- day 4 samples from Fig. EV6C is shown. (**E**) PCA of RNA-seq results from this study; naïve (J1), AKO, DKO, and GKO mESCs and samples from GSE131556 (FS1, FS2, AF, AFX, E5, E5.5, E6) are shown. To induce formative status, Activin A (A), Fgf2 (F) or iWnt (X) is treated in the study GSE131556. Representative formative status samples are marked with dotted circle in the plot. (**F**) PCA from RNA-seq of mESCs in this study (naïve (J1), AKO, GKO and DKO) with diapause-like mESCs from GSE143492 (ICM, dICM, Epi). Diapause-like cells are marked with dotted circle in the plot. (**G**) T7E1 assay of *Prkaa1* gRNA target site for WT, GKO and DKO in OG-ESCs (left panel), sequence information of targeted *Prkaa1* from WT, GKO and DKO in OG-ESCs (right panel). Asterisks indicate the observed bands corresponding to expected size after T7E1 treatment. (**H**) Immunoblotting analysis with OG-WT, OG-GKO and OG-DKO mESCs with indicative proteins (pACC, total ACC, pAMPKα, total AMPK), β-actin is used as an internal loading control of the analysis. OG-ESCs: OG2$^{+/-}$GOF6$^{+/-}$ ESCs. (**I**) Representative brightfield images for Cont, GKO and DKO OG-ESCs (scale bar = 200 μm). (**J**) Fold mRNA expressions for *Esrrb*, *Rex1* and *Klf4* in WT, GKO and DKO OG-ESCs. Two-way ANOVA, multiple comparisons, (****$P < 0.0001$, ***$P = 0.0005$, $N = 3$, $n = 5$). OG-ESCs: OG2$^{+/-}$GOF6$^{+/-}$ ESCs.

 © The Author(s)
